# Strigolactone synthesis is ancestral in land plants, but canonical strigolactone signalling is a flowering plant innovation

**DOI:** 10.1186/s12915-019-0689-6

**Published:** 2019-09-05

**Authors:** Catriona H. Walker, Karen Siu-Ting, Alysha Taylor, Mary J. O’Connell, Tom Bennett

**Affiliations:** 1 School of Biology, Faculty of Biological Sciences, University of Leeds, Leeds, LS2 9JT UK; 2 Institute for Global Food Security, School of Biological Sciences, Queens University, Belfast, BT7 1NN UK; 3 Computational and Molecular Evolutionary Biology Research Group, School of Biology, Faculty of Biological Sciences, University of Leeds, Leeds, LS2 9JT UK; 4 Computational and Molecular Evolutionary Biology Research Group, School of Life Sciences, Faculty of Medicine and Health Sciences, University of Nottingham, Nottingham, NG7 2RD UK; 5 Institute of Biological, Environmental and Rural Sciences, Aberystwyth University, Aberystwyth, SY23 3FD UK

**Keywords:** Strigolactones, Strigolactone synthesis, Strigolactone signalling, Phylogenetics

## Abstract

**Background:**

Strigolactones (SLs) are an important class of carotenoid-derived signalling molecule in plants, which function both as exogenous signals in the rhizosphere and as endogenous plant hormones. In flowering plants, SLs are synthesized by a core pathway of four enzymes and are perceived by the DWARF14 (D14) receptor, leading to degradation of SMAX1-LIKE7 (SMXL7) target proteins in a manner dependent on the SCF^MAX2^ ubiquitin ligase. The evolutionary history of SLs is poorly understood, and it is not clear whether SL synthesis and signalling are present in all land plant lineages, nor when these traits evolved.

**Results:**

We have utilized recently-generated genomic and transcriptomic sequences from across the land plant clade to resolve the origin of each known component of SL synthesis and signalling. We show that all enzymes in the core SL synthesis pathway originated at or before the base of land plants, consistent with the previously observed distribution of SLs themselves in land plant lineages. We also show that the late-acting enzyme LATERAL BRANCHING OXIDOREDUCTASE (LBO) may be considerably more ancient than previously thought. We perform a detailed phylogenetic analysis of SMXL proteins and show that specific SL target proteins only arose in flowering plants. We also assess diversity and protein structure in the SMXL family, identifying several previously unknown clades.

**Conclusions:**

Overall, our results suggest that SL synthesis is much more ancient than canonical SL signalling, consistent with the idea that SLs first evolved as rhizosphere signals and were only recruited much later as hormonal signals.

**Electronic supplementary material:**

The online version of this article (10.1186/s12915-019-0689-6) contains supplementary material, which is available to authorized users.

## Background

The ability to tailor growth and development to prevailing environmental conditions is a key feature of plant biology and has been instrumental in the successful colonization of all terrestrial biospheres by plants. Plant growth is coordinated in space and time through the production and systemic transport of plant hormones; external modulation of these signals allows coupling of environment and development. Strigolactones (SLs) are a class of carotenoid-derived signalling molecules which function endogenously as hormones, while also acting in an exogenous manner as signals in the rhizosphere (reviewed in [1]). SLs play a key role in multiple developmental pathways, including the regulation of shoot branching, lateral root formation and leaf growth. Additionally, the exudation of SLs from the roots into the soil has been shown to be a key factor for the recruitment of arbuscular mycorrhizal (AM) fungi [2]. SLs are particularly associated with soil phosphate levels, SL synthesis is upregulated in low phosphate conditions [3] and the subsequent recruitment of AM fungi provides the plant with phosphate in exchange for reduced carbon. A proportion of the SLs synthesized within the root are transported into the shoot system, where an inhibitory effect on shoot branching allows the plant to modify shoot system size in direct relation to the availability of soil-borne resources [4].

In flowering plants (angiosperms), the synthesis of SLs is carried out by a core pathway of four enzymes, which have been characterized in multiple species (reviewed in [1]). The initial substrate all-trans-β-carotene is processed by the carotene isomerase DWARF27 (D27) to 9-cis-β-carotene [5], which is subsequently cleaved and modified by two carotenoid cleavage dioxygenases (CCD7 and CCD8) in turn [5]. The resulting product, carlactone (CL), is the common precursor for all known SLs, but must be modified by cytochrome P450 enzymes of the MAX1 family to form carlactonoic acid (CLA) or other active derivatives [6, 7]. These intermediates are thought to be further processed by an array of enzymes that result in a diverse set of active SL structures (e.g. [8]). In *Arabidopsis*, LATERAL BRANCHING OXIDOREDUCTASE (LBO) has been identified as late-acting enzyme that converts CLA to methyl-CLA (MeCLA), but it assumed further enzymes must also exist, as MeCLA is not an abundant naturally occurring SL in *Arabidopsis* [8]. SL signalling is mediated by the DWARF14 (D14) α/β hydrolase receptor, which can both bind and hydrolyse SLs; the relative importance of hydrolysis in signalling is still an open question [9–12]. SL binding triggers a conformational change in D14 that mediates its interaction with MAX2, an F-box protein that forms part of an SCF ubiquitin ligase complex, which targets proteins for proteolytic degradation [9–11]. The target proteins of D14 are members of the HSP101-like SMAX1-LIKE family, specifically the SMAX1-LIKE7/DWARF53 (SMXL7/D53) sub-family. Recruitment of SMXL7 proteins to the signalling complex by active D14 results in the ubiquitination and subsequent degradation of both the D14 and SMXL proteins [13–16]. Turnover of SMXL7 proteins allows downstream SL responses to occur, which seem to include both removal of the PIN1 auxin efflux carrier from the plasma membrane of cells in the stem [15, 17, 18] and increased transcription of BRANCHED1-type transcription factors [15, 16, 19, 20]. SMXL proteins are not DNA-binding transcription factors, but have a well-conserved ERF-associated repressive (EAR) motif, and have thus been proposed to act as intermediates in the assembly of repressive transcriptional complexes, via recruitment of TOPLESS family chromatin remodelling complexes [21]. There is some evidence for this, particularly in rice [22], but generally transcriptional responses to SL are limited [23], and the EAR motif is not absolutely required for SMXL7 function [17]. The function of SMXL proteins thus remains rather enigmatic, and it is possible that they have multiple cellular functions, both transcriptional and non-transcriptional.

The evolution of SL synthesis and signalling has generated equal amounts of interest and confusion. It is clear that this evolution history is not simple, with different components appearing at different points in the evolutionary record [1]. For instance, with regard to SL synthesis, it has been proposed that D27 arose in the algal ancestors of land plants, CCD7 at the base of the land plant group, CCD8 after the divergence of liverworts and other land plants, MAX1 within the vascular plant group and LBO specifically within seed plants [1, 8, 24, 25]. Outside flowering plants, SL synthesis has been characterized in the moss *Physcomitrella patens*, where CCD7 and CCD8 act consecutively in CL synthesis as in angiosperms [26, 27]. There is some uncertainty about which strigolactones are ultimately synthesized by *P. patens*, with recent analysis suggesting only CL is produced, consistent with the lack of MAX1 orthologue in this species [27, 28]. In general, conclusions regarding SL synthesis outside the angiosperms are based on very limited sampling of sequences. Conversely, a more exhaustive approach to sampling has recently demonstrated that SL signalling via canonical D14-type SL receptors appears to be a relatively recent innovation within the seed plants [29]. Understanding the evolution of SL signalling is complicated by the apparent origin of the signalling pathway through duplication of an existing pathway. D14 proteins are closely related to the KARRIKIN-INSENSITIVE2 (KAI2) sub-family of α/β hydrolases and appear to have arisen by duplication of *KAI2* near the base of land plants followed by gradual neo-functionalization [29]. KAI2-like proteins are found in charophyte algae, indicating a very ancient origin for KAI2 itself [29]. In angiosperms, KAI2 acts in the perception of smoke-derived karrikin molecules in the environment, but it is also assumed to act as a receptor for an as-yet-unidentified endogenous compound (KAI2-Ligand, KL) (reviewed in [1]). Both D14 and KAI2 signalling act through SCF^MAX2^ [30]; MAX2 itself has an ancient origin in the algal ancestors of land plants [29]. SMXL7 proteins are also closely related to the presumptive targets of KAI2 signalling, members of the SUPPRESSOR OF MAX2 1 (SMAX1) sub-family of the SMXL family [31, 32], and it has recently been suggested that the SMXL7 sub-family may also be a relatively recent innovation in plants [33].

The evolution of SLs thus represents something of enigma, but the evidence is currently highly fragmentary, and based on limited sampling from non-representative genomes. In order to try and unravel this mystery, we have exploited recently generated genomic and transcriptomic sequences from across the land plant clade, to reassess the distribution and evolutionary history of synthesis and signalling components in land plants.

## Results

### Identification and analysis of SL synthesis and signalling genes

In order to understand the evolution of SL synthesis and signalling with greater resolution, we obtained 450 sequences from 153 species, covering all the major land plant groups, charophyte algae and where appropriate chlorophyte algae (summarized in Additional file 1). We performed phylogenetic analyses on the retrieved sequences using both Bayesian and maximum likelihood (ML) methodologies, at both nucleotide and amino acid levels. We used these analyses to understand the evolution of each strigolactone-related gene family in turn.

### D27 is ancestral in land plants

It was previously suggested that D27-like proteins are found in both chlorophyte and charophyte algae [24, 34], implying that D27 proteins should be present in all land plant groups. We identified unambiguous *D27* genes from the fully sequenced genomes of many angiosperms, *Selaginella moellendorfii*, *Physcomitrella patens*, *Sphagnum fallax* and *Marchantia polymorpha*. We obtained sequences similar to *D27* from transcriptomic datasets for all major taxa, with the exception of hornworts. However, in preliminary phylogenetic analyses, none of these transcriptome sequences grouped with the set of *D27* sequences from completed genomes. We thus reciprocally BLASTed these new ‘*DWARF27-LIKE1*’ (*D27L1*) sequences against fully sequenced genomes, and from each, we identified a gene that was more closely related to *D27L1* than *D27*. We thus added these additional *D27L1* sequences to our dataset. We identified sequences from various algal genomes with similarity to *D27*/*D27L1*, which were included in our alignments (Additional file 2). Finally, we retrieved sequences from *Arabidopsis*, rice and *Amborella trichopoda* that are more distantly related to D27 (*D27L2*), for use as a possible outgroup.

**Fig. 1:**
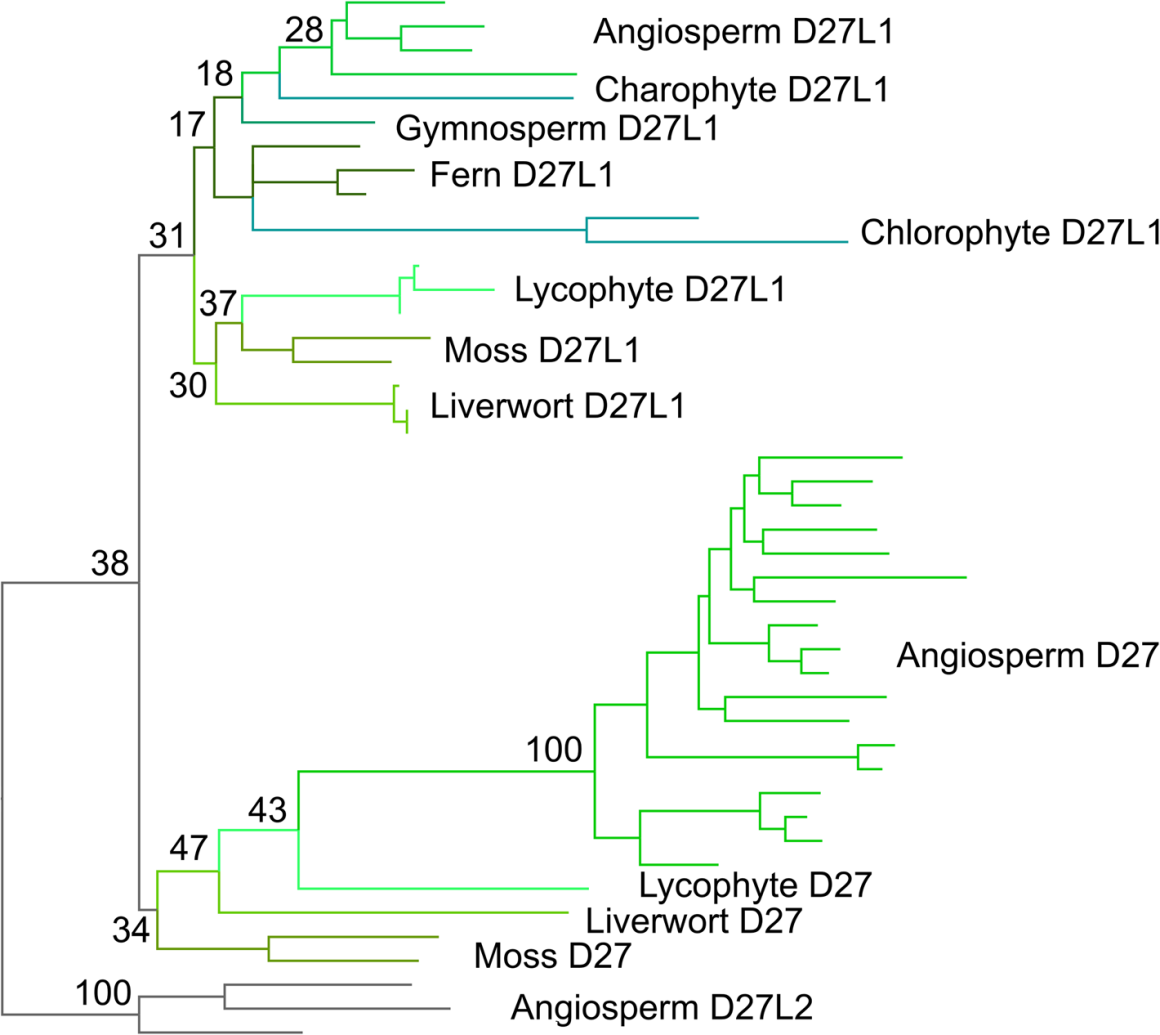
Optimal phylogeny for D27 family. Maximum likelihood (ML) tree under the MGK+F3X4+R5 codon model in IQtree. Topology rooted with the *D27L2* clade. Key bootstrap values are shown along the backbone of the tree. The fully labelled phylogeny is shown in Additional file 3A

Full phylogenetic analysis of the final sequence dataset generated three topologies from the nucleotide and amino acid alignments (Additional file 3A-C; Table 1). Trees were rooted using the *D27L2* clade, which was monophyletic in all analyses. All analyses grouped *D27L1* sequences into a single clade, which in each case included the algal *D27*-like sequences. The algal sequences were not monophyletic and appeared misplaced within the *D27L1* clade. We were not able to recover the monophyly of *D27* using the amino acid dataset with both BI and ML methods, with the moss and lycophyte sequences taxa not clustering with the rest of the sequences. However, this issue was resolved when using codon models, which are able to retrieve the monophyly of *D27*, albeit with low node support (Fig. 1). The Approximately Unbiased test (AU test) based on the nucleotide dataset rejected the amino acid ML and BI trees, thus indicating that the ML tree based on codon models is the most optimal topology (Fig. 1; Table 1).

**Table 1:** Summary of hypothesis testing results for the six gene families tested

Dataset	Tree	logL	*p*-WKH	*p*-WSH	*p*-AU
D27	Phylobayes_tree_AA	− 29,047.235	0.0037	0.0056	0.0024
D27	Raxml_tree_AA	− 29,033.177	0.0181	0.0351	0.017
D27	IQ_tree_Nucl	− 28,989.821	0.9819	0.9901	0.9872
LBO	Phylobayes_tree_AA	− 14,392.989	0.0619	0.104	0.0339
LBO	Raxml_tree_AA	− 14,384.488	0.938	0.997	0.971
LBO	IQ_tree_Nucl	− 14,454.098	0.0008	0.0009	0.000624
MAX1	Phylobayes_tree_AA	− 11,620.825	0.0033	0.0062	0.0007
MAX1	Raxml_tree_AA	− 11,556.029	0.8381	0.9544	0.848
MAX1	IQ_tree_Nucl	− 11,570.886	0.1619	0.2706	0.1579
CCD7	Phylobayes_tree_AA	− 15,752.52	0.1431	0.2475	0.16
CCD7	Raxml_tree_AA	− 15,742.717	0.8569	0.9674	0.9133
CCD7	IQ_tree_Nucl	− 15,785.588	0.0353	0.047	0.0359
CCD8	Phylobayes_tree_AA	− 11,524.537	0.0427	0.0642	0.0205
CCD8	Raxml_tree_AA	− 11,514.983	0.957	0.998	0.981
CCD8	IQ_tree_Nucl	− 11,621.601	0.0013	0.0015	0.0000293
SMXL	Phylobayes_tree_AA	− 62,476.375	0.0058	0.0106	0.0077
SMXL	Raxml_tree_AA	− 62,393.986	0.9942	0.9992	0.9923
SMXL	RAxML_tree_Nucl	− 62,710.892	0	0	0

Loglikelihood (logL) per topology, as well as *p* value for weighted KH test (*p*-WKH); *p* value for weighted SH test (*p*-WSH) and *p* value for the Approximately Unbiased test (*p*-AU) as calculated in IQ-tree. Highlighted in grey are the non-rejected hypotheses. See the ‘Materials and methods’ section for further details

For our analyses, it seems clear that the *D27* and *D27L1* sequences form two coherent clades, present across all land plants (Fig. 1); the distinction between the clades is also evident in the markedly different N-terminal protein sequences (Additional file 2). Our results are highly congruent with the phylogeny presented in [35], in which 3 coherent clades of D27-like proteins were identified in land plants, albeit with much lower sampling depth. Our failure to identify any new *D27* sequences from transcriptome assemblies (while recovering abundant *D27L1*sequences) is intriguing and suggests *D27* is either expressed at very low levels, or in a spatially restricted manner in the non-flowering plant species. A clear unresolved question from our analyses is whether the *D27*-*D27L1* split occurred in the charophyte algae, or at the base of land plants. The few algal sequences we obtained group with *D27L1* sequences, suggesting they are more closely related at a sequence to level to *D27L1*. Based on this tentative evidence, and comparison of protein sequences between algal D27-like sequences, *D27* and *D27L1* (Additional file 2), we hypothesize that *D27* represents a neo-functional lineage in land plants derived by duplication of the ancestral *D27L1* lineage at the base of land plants. Further resolution of this question awaits improved availability of genome sequences from algal species.

### CCD7 is ancestral in land plants

We identified *CCD7*-like sequences from chlorophytes, charophytes and the major land plant groups, with the exception of hornworts and monilophytes (Additional file 4). Phylogenetic analysis of the broader *CCD* family shows these sequences form a monophyletic group (Additional file 5, Additional file 6). The lack of sequences from monilophytes is somewhat surprising, but in general, we only identified 6 *CCD7* sequences from transcriptome assemblies, suggesting that *CCD7* expression is generally low or spatially restricted in land plants and that the lack of sequences from monilophytes is likely a sampling error.

**Fig. 2:**
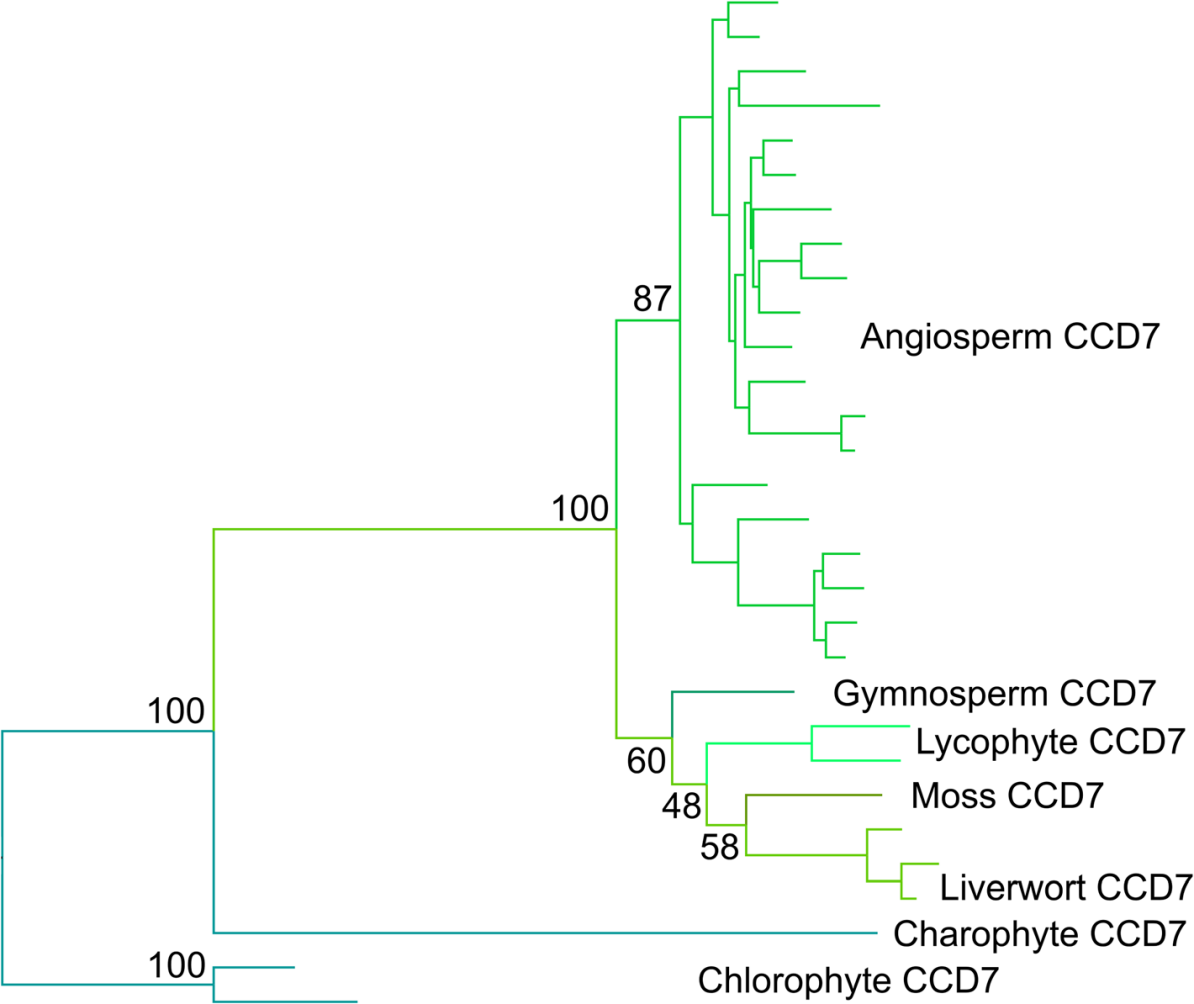
Optimal phylogeny for CCD7 family. Maximum likelihood (ML) tree with the amino acid dataset under the PROTCATLGX model in RAxML. Topology rooted with the chlorophyte *CCD7* clade. Key bootstrap values are shown along the backbone of the tree. The fully labelled phylogeny is shown in Additional file 7C

Full phylogenetic reconstructions of the final sequence dataset for *CCD7* are reported in Additional file 7A-C. Amino acid datasets retrieve very similar topologies with both ML and BI, with very similar support values. The only difference in the topology is in the relationships of some of the tips, particularly internal nodes within the grasses (Poaceae). The nucleotide dataset using codon models gave a similar topology to the amino acid dataset, but misplaced the grass CCD7 sequences as sister to all other land plant sequences. Overall, support values for this topology are lower than the trees retrieved with the amino acid alignment, and the AU test based on the amino acid dataset only rejected the nucleotide ML codon-model tree. The ML and BI amino acid trees were equally likely, but based on the overall log-likelihood, the ML amino acid tree seemed to be the most optimal topology (Fig. 2; Table 1). This was also the topology most consistent with established organismal phylogeny among land plants.

Our analyses thus suggest the evolutionary history of *CCD7* is relatively simple, with no major duplications present in the family, implying a strong pressure to maintain *CCD7* as a single copy gene. The CCD7 lineage clearly predates the evolution of land plants, and unambiguous CCD7 sequences are found throughout the land plant clade. Delaux et al. [24] showed that the algal CCD7 sequences are quite distinct from land plant sequences and that the proteins may have rather different substrate specificity, so it is probable that ‘true’ CCD7 activity is only found in land plants.

### CCD8 is ancestral in land plants

Previous work has suggested that *CCD8* is absent from liverworts, leaving questions open as to the origin of the *CCD8* lineage [1]. However, while *CCD8* is indeed absent from the completed genome of *Marchantia polymorpha*, we obtained multiple unambiguous *CCD8* sequences from other liverworts; *M. polymorpha* is thus an exception, rather than the rule. Indeed, we obtained unambiguous *CCD8* sequences from all the major land plant groups (including hornworts), and *CCD8*-like sequences from chlorophyte algae (Additional file 8); phylogenetic analysis shows that these are all part of a monophyletic clade relative to the wider CCD family (Additional file 6).

**Fig. 3:**
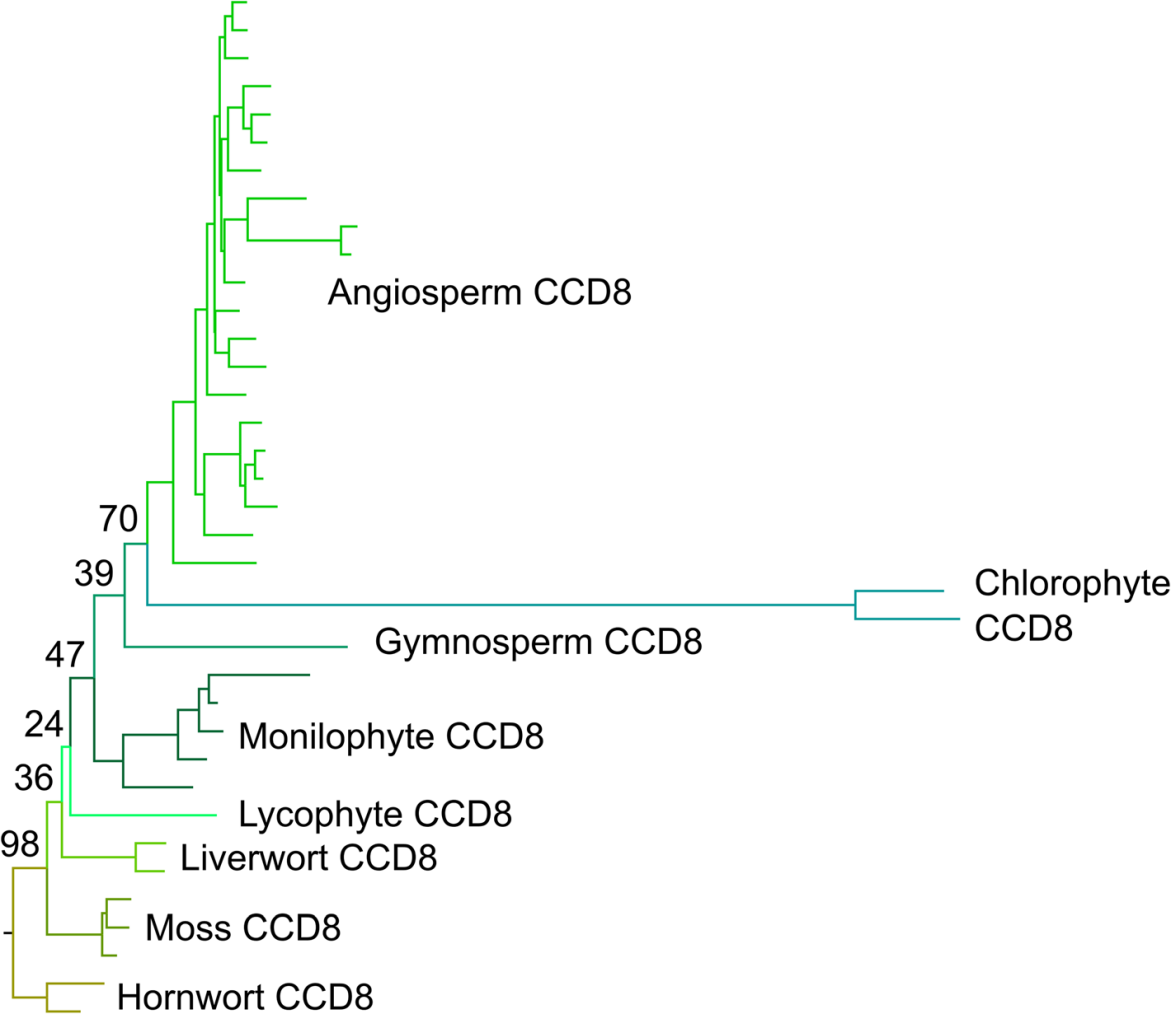
Optimal phylogeny for CCD8 family. Maximum likelihood (ML) tree with the amino acid dataset under the PROTCATLGX model in RAxML. Topology rooted with the hornwort CCD8 clade. Key bootstrap values are shown along the backbone of the tree. The fully labelled phylogeny is shown in Additional file 9C

Full phylogenetic reconstructions of the final sequence dataset for *CCD8* are reported in Additional file 9A-C. If rooted with the chlorophyte algal sequences, the three resulting topologies were very different. However, if rooted with the hornwort sequences, the resulting topologies were very similar, with the branching order of clades following well-established organismal phylogeny in land plants. The only major difference between the topologies was the placing of the chlorophyte sequences as erroneous in-groups at the base of the angiosperms, or within the Poaceae. The Approximately Unbiased test (AU test) based on the amino acid dataset rejected the amino acid Bayesian tree and the nucleotide ML tree with codon models, thus indicating that the amino acid ML tree was the most optimal topology (Fig. 3, Table 1).

As with *CCD7*, our analyses suggest the evolutionary history of *CCD8* is relatively simple, with no major duplications present in the family, implying a strong pressure to maintain *CCD8* as a single copy gene. The CCD8 lineage also predates the evolution of land plants (Additional file 6), and unambiguous CCD8 sequences are found throughout the land plant clade. This misplacement of the algal sequences in the phylogeny suggests that, as for CCD7, the algal CCD8 sequences are quite distinct from land plant sequences and that the proteins likely have rather different substrate specificity.

### MAX1 is ancestral in land plants

**Fig. 4:**
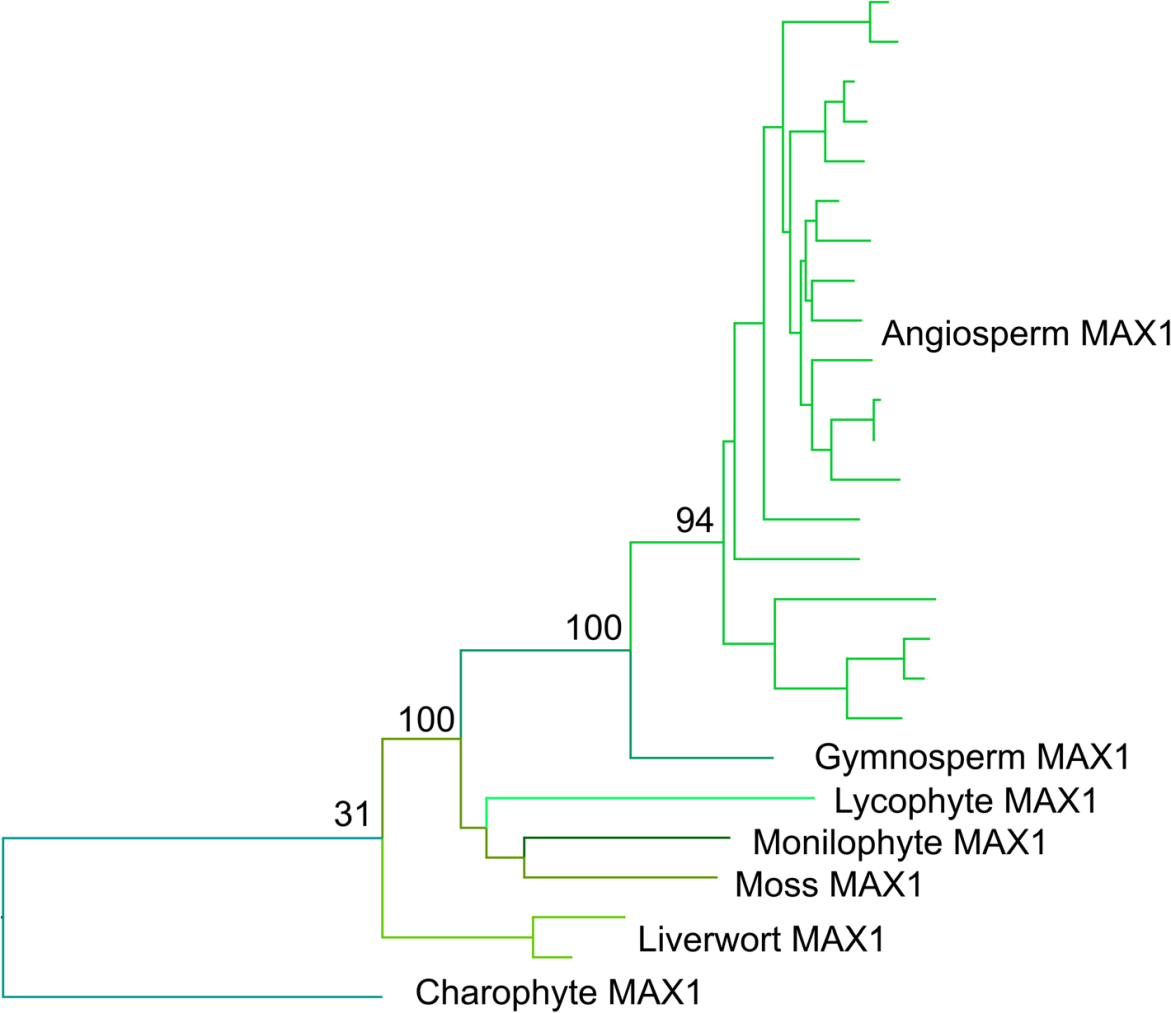
Optimal phylogeny for MAX1 family. Maximum likelihood (ML) tree with the amino acid dataset under the PROTCATLGX model in RAxML. Topology rooted with the algal MAX1 clade. The fully labelled phylogeny is shown in Additional file 11C

The evolutionary history of the MAX1 family was previously surveyed by [25], but no *MAX1*-like sequence was identified in *P. patens*, leading to uncertainty regarding the evolutionary origin of the *MAX1* function in SL synthesis. We identified unambiguous MAX1 sequences from all major land plant groups except hornworts, and a *MAX1*-like sequence from *Klebsormidium nitens*, though we did not obtain any sequences from chlorophyte algae (Additional file 10). This suggests a much earlier origin of *MAX1* than was previously apparent. While *P. patens* does indeed have no *MAX1*, other mosses possess copies of *MAX1*, as do most liverworts—although as with *CCD8*, *M. polymorpha* does not (Fig. 4). As with CCD7 and CCD8, there are no major duplications in the *MAX1* family, which is present as a single copy gene in most species.

Full phylogenetic reconstructions of the final sequence dataset for *MAX1* are reported in Additional file 11A-C. The three resulting topologies agreed in the retrieval of a split that separated seed plants from non-seed plants. However, most of the placement of the internal nodes disagreed among the three topologies. In addition to this, the nucleotide and amino acid ML trees seemed to be the most similar in topology, while the BI gave the most distinct. The Approximately Unbiased test (AU test) based on the amino acid dataset only rejected the amino acid BI tree. Either ML in amino acid and nucleotide trees are equally likely, but based on the overall log-likelihood, the ML amino acid tree presented the most optimal topology (Fig. 4, Table 1). This was also the topology most consistent with organismal phylogeny among land plants.

### LBO-like proteins are found throughout land plants

Based on a relatively simple phylogeny, Brewer et al. [8] concluded that since LBO-like proteins were not present in *Physcomitrella patens* and *Selaginella moellendorfii*, LBO likely represented a seed plant innovation. However, this approach used two non-representative genomes to make conclusions regarding all non-seed plants. We reinvestigated the evolution of *LBO* using a broad sampling approach and identified a clade of proteins present in all land plants, which contained *Arabidopsis* LBO (Additional file 12). This clade contained the previously described DOXC53 (LBO) and uncharacterized DOXC54 angiosperm clades and equivalent sequences from gymnosperms [8]. We named the DOXC54 clade RELATED TO STRIGOLACTONE SYNTHESIS (RSS).

Full phylogenetic reconstructions of the final sequence dataset for *LBO* are reported in Additional file 13A-C. It was challenging to reconstruct the evolution of this gene family, and the models and methods tested in both amino acid and nucleotide datasets do not generate congruent topologies. Furthermore, none of the topologies are entirely consistent with organismal phylogeny. However, some general features can be observed. In all topologies, a monophyletic eu-LBO clade containing angiosperm and gymnosperm sequences is recovered (Additional file 13A-C). The two amino acid trees placed the proto-LBO (p-LBO) sequences from hornworts, liverworts, mosses and lycophytes as a basal grade with respect to all other RSS/LBO sequences (Additional file 13A-B). In addition to the monopyletic eu-LBO clade, the two amino acid trees also recover a monophyletic group of RSS sequences from angiosperms (the previously described DOX54 clade), a monophyletic group of sequences from gymnosperms and a monophyletic group of sequences from ferns. However, the relative arrangement of these clades varies.

**Fig. 5:**
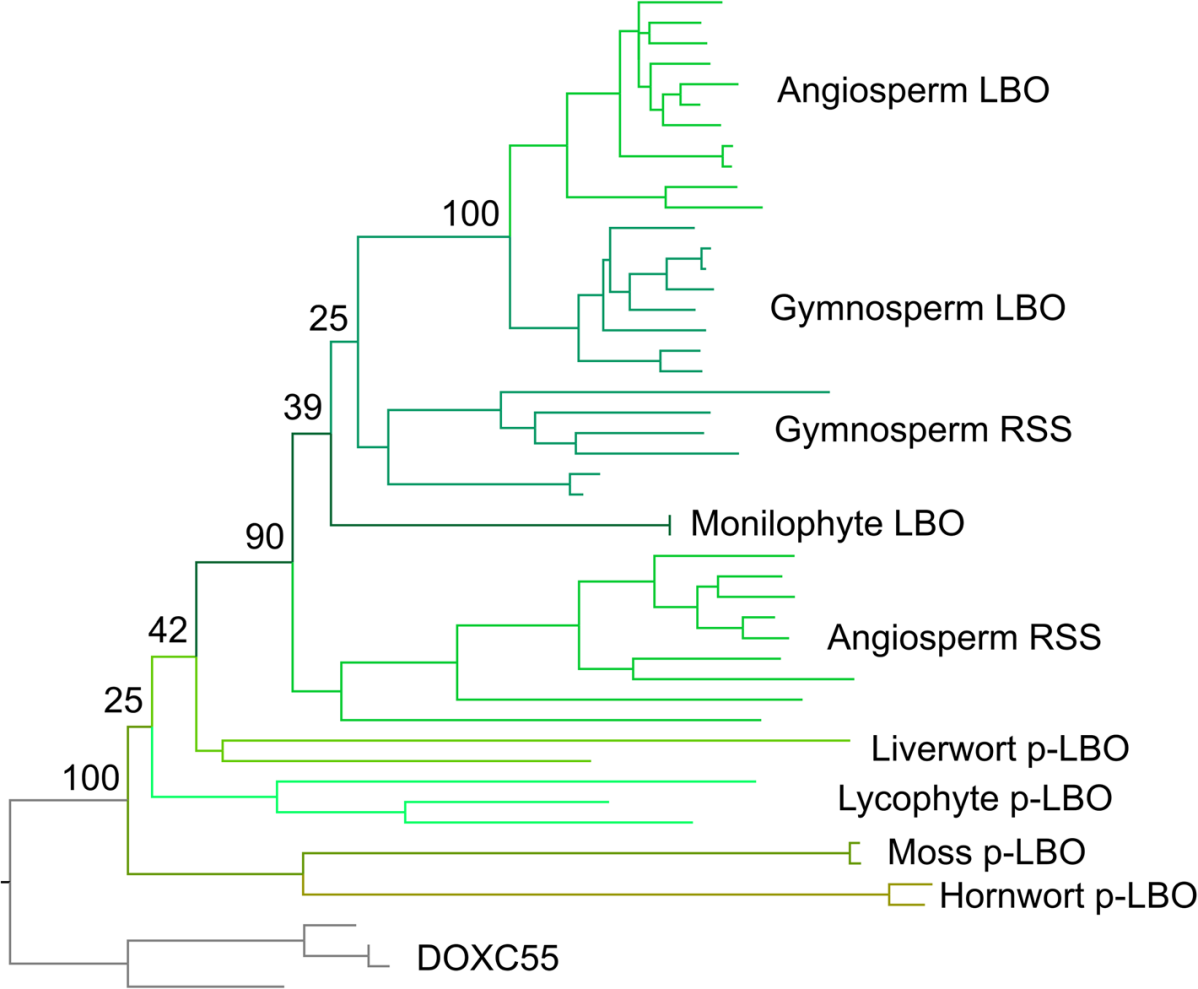
Optimal phylogeny for LBO family. Maximum likelihood (ML) tree with the amino acid dataset under the PROTCATLGX model in RAxML. Topology rooted with the DOXC55 clade. The fully labelled phylogeny is shown in Additional file 13A

The Approximately Unbiased test based on the amino acid dataset rejected both the amino acid BI tree and the nucleotide ML tree, suggesting the amino acid ML tree is the most optimal topology. In this tree, angiosperm RSS is sister to a clade containing the fern sequences, the second gymnosperm clade, and the eu-LBO clade. However, the most parsimonious explanation for the evolution of this family would appear to be that there was duplication in the ancestral proto-LBO lineage at the base of seed plants, giving rise to separate RSS and LBO clades that are present in both angiosperms and gymnosperms, and that the monilophyte clade is misplaced in this analysis (Fig. 5). Brewer et al. [8] suggested that LBO activity arose in seed plants, which would imply the *LBO* lineage has neo-functionalized after this hypothetical duplication. However, there is no evidence for this model in the nucleotide or protein sequence data, in which both RSS and LBO proteins are equally similar to proto-LBO proteins non-seed plants (Additional file 12). Similarly, the branch lengths of the RSS and LBO sub-families give no indication of evolutionary innovation in either lineage (Fig. 5). Thus, we propose it is likely that RSS and LBO both retain the ancestral function found in the proto-LBO lineage in non-seed plants.

### SMXL proteins are present throughout land plants but not in algae

We next turned our attention to understanding the evolution of SL signalling. We have recently examined the evolution of SL receptors in the *D14*/*KAI2* family and shown that SL perception probably evolved gradually by neo-functionalization of KAI2-like receptors [29]. We also showed that MAX2 is a deeply conserved protein in charophyte algae and land plants [29]. However, little is known regarding the evolution of the SMXL family proteins that are the proteolytic targets of SL and KL signalling, although a recent report has provided significant insights into SMXL evolution within seed plants [33]. In order to understand *SMXL* evolution, we obtained 223 *SMXL* sequences from 99 species (Additional file 14). We identified unambiguous *SMXL* sequences in all major land plant groups, but not in any of the chlorophyte or charophyte genome/transcriptome datasets (Table 2). Preliminary phylogenetic analyses indicated a more complex evolutionary history than the SL synthesis enzymes and placed *SMXL* family members into clear taxon-specific clades (Table 2).

**Table 2:** Major clades in the *SMXL* family

Clade	Taxon	Sub-taxon	Sequences	Major sub-clades
*SMXLA*	Liverworts		5	
*SMXLB*	Mosses		19	*SMXLB*
		Bryopsida	10	*SMXLC*
*SMXLD*	Hornworts		6	
*SMXL*	Lycophytes	Lycopodiales	6	*SMXLE*
		Isoetales	2	*SMXLF*
		Selaginellales	7	*SMXLG*
		Selaginellales	6	*SMXLH*
*SMXLJ*	Monilophytes		17	
*SMXL4*				
*gSMXL4*	Gymnosperms		5	
*aSMXL4*	Angiosperms		25	
*SMXL39*	Angiosperms		8	*SMXL39*
		Core eudicots	16	*SMXL3*
		Core eudicots	13	*SMXL9*
*SMAX1*				
*gSMAX1*	Gymnosperms		14	
*aSMAX1*	Angiosperms		31	
*SMXL78*	Angiosperms		7	*SMXL78*
		Core eudicots	16	*SMXL7*
		Core eudicots	13	*SMXL8*

Table showing major clades in the *SMXL* family, as defined at the level of major taxonomic groups. Almost all sequences in the family unambiguously group into one of these clades. Within some clades, there are major sub-clades where the lineage has been duplicated; these are listed at the right. Our analysis suggests that seed plant *SMXL* proteins group into two super-clades, *SMAX1* and *SMXL4*, as indicated on the left of the table

We identified 1 *SMXL* clade in each of the liverworts (*SMXLA*), mosses, hornworts (*SMXLD*), lycophytes and monilophytes (*SMXLJ*) (Table 2). In mosses of the Bryopsida, there are two distinct *SMXL* sub-clades (*SMXLB* and *SMXLC*), but only a single *SMXL* clade (resembling *SMXLB*) is present in the early-diverging Sphagnopsida lineage. These results are consistent with the evolution of the *D14*/*KAI2* family in mosses, where four *D14*/*KAI2* sub-clades are present in the Bryopsida, but only two in the Sphagnopsida [29]. Collectively, these data suggest that whole-genome duplication may have occurred at the base of the Bryopsidan lineage. Similarly, in the lycophyte group, there are two *SMXL* sub-clades present in the Selaginellales (*SMXLG* and *SMXLH*), but only one in the Lycopodiales (*SMXLE*) and Isoetales (*SMXLF*). Although they form a monophyletic lycophyte clade, there is little resemblance between SMXLE, SMXLF, SMXLG or SMXLH proteins. This degree of sequence divergence within the lycophytes is consistent with our previous observations of D14/KAI2 and PIN protein family members [29, 36]. We detected two distinct clades of *SMXL* proteins in gymnosperms and at least four distinct clades in angiosperms (Table 2).

### Diversification of SMXL proteins in the seed plant lineage

**Fig. 6:**
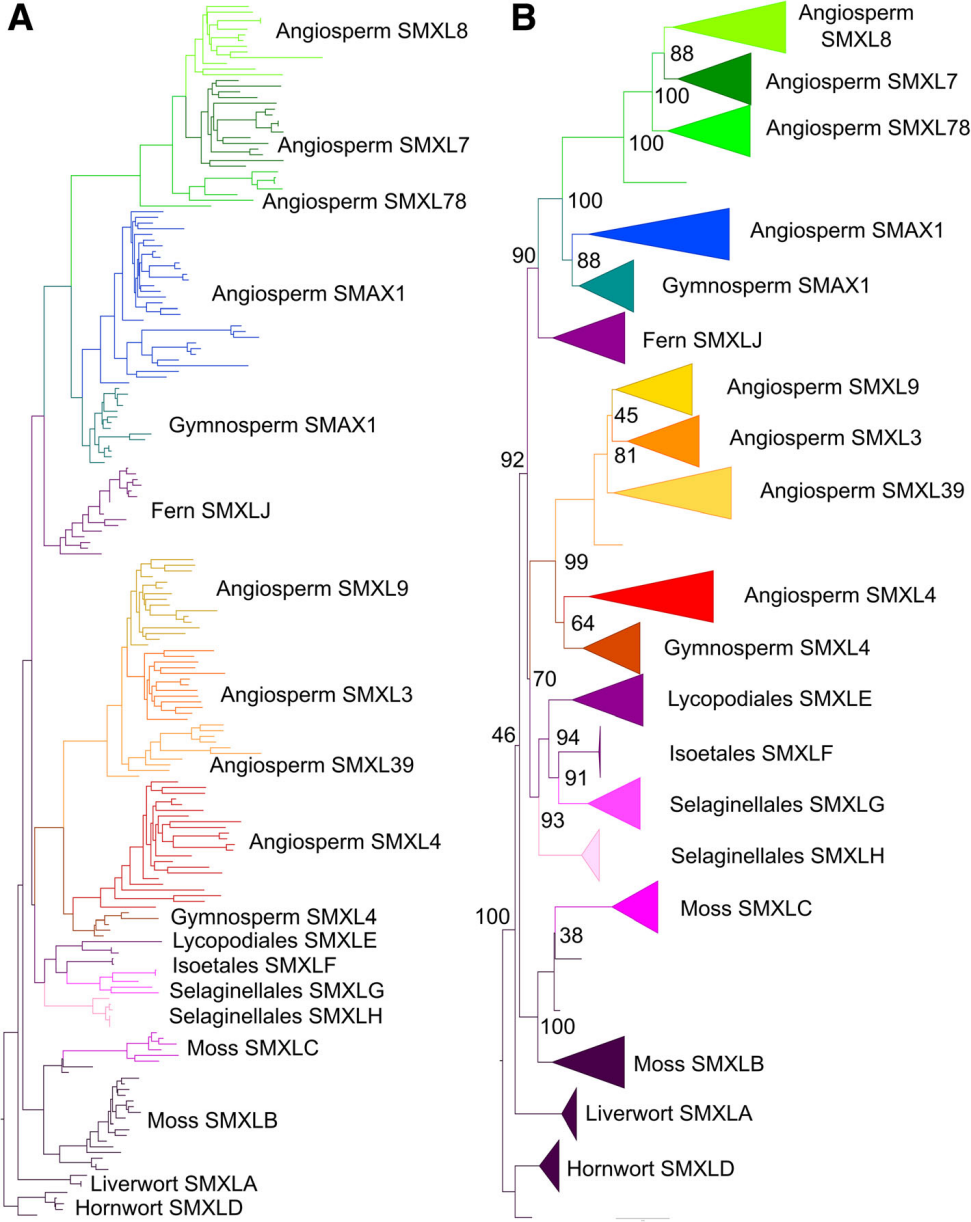
Optimal phylogeny for SMXL family. Maximum likelihood (ML) tree with the amino acid dataset under the PROTCATJTTX model in RAxML. Topology rooted at the hornwort clade. **a** Phylogram, labelled to show the high-order relationships between the major clades. **b** Cladogram showing more detailed relationships between the clades listed in Table 2, with bootstrap values at key nodes. The fully labelled phylogeny is shown in Additional file 17

To understand the interrelationship of these clades, we reconstructed the evolution of the family. Full phylogenetic reconstructions of the final sequence dataset for SMXL are reported in Additional files 15, 16 and 17. The three topologies were broadly congruent with each other in the reconstruction of the SMXL gene family. The SMXL alignment in amino acid format failed the likelihood mapping test, while the trimmed amino acid and nucleotide alignments for this gene family passed our likelihood mapping analyses, but they were unable to reject alternative topologies. The Approximately Unbiased test (AU test) based on the amino acid alignment rejected the nucleotide ML and amino acid BI trees, thus indicating that the amino acid ML tree is the optimal topology (Fig. 6, Table 1). The amino acid ML tree also most closely recapitulates organismal phylogeny, so we chose this as the optimal tree. In the absence of an obvious algal outgroup, we used liverwort *SMXL* sequences to root the trees, consistent with the traditional view of land plant phylogeny [37]. More recent analyses have suggested hornworts might be the earliest diverging land plant lineage [38], and we are also able to root the tree with hornwort sequences without altering the topology of the tree.

**Fig. 7:**
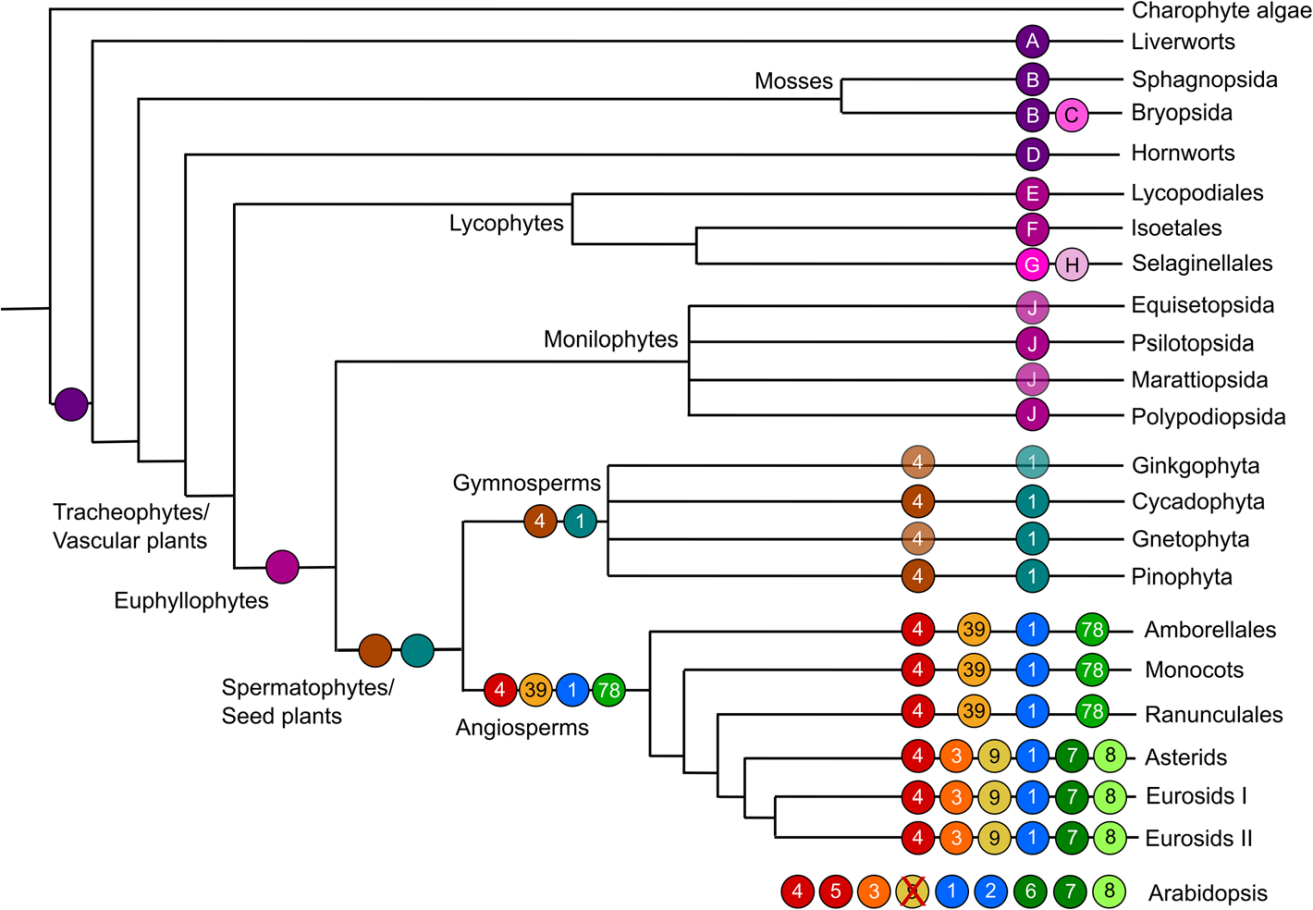
Reconstruction of SMXL evolution. Schematic depicting the complement of SMXL proteins in major land groups, and their inferred evolutionary origin. Each branch indicates a major land plant group; lycophytes, monilophytes and gymnosperms are further sub-divided into relevant orders/families/etc. The circles on each branch indicate the core complement of proteins in that group or sub-group. Clades which are inferred by parsimony are denoted with a translucent circle, and clades believed to have been lost are shown with a red cross. Letters and numbers in the circles indicate clade names. Circles without symbols at internal branching points represent the minimum inferred SMXL protein complement in the last common ancestor of each major land plant group

All analyses confirm the monophyly of the major clades in Table 2 and suggest a basic topology for the *SMXL* family. Consistent with both long-held and current notions of organismal phylogeny in land plants, the liverwort, moss and hornwort *SMXL* clades are arranged as grade with respect to a large clade containing the tracheophyte sequences (Fig. 6). Sequences from seed plants grouped into two super-clades which we denote as *SMAX1* and *SMXL4*. In our reconstruction, the lycophyte *SMXL* sequences grouped with seed the plant *SMXL4* super-clade, while the monilophyte *SMXL* sequences grouped with the seed plant *SMAX1* super-clade (Fig. 6). This is congruous with the phylogeny presented by [33]. A strict reading of this phylogeny would imply a duplication at the base of vascular plants, followed by loss of one clade each in lycophytes and monilophytes. However, the most parsimonious explanation is that there was a duplication at the base of seed plants and that the single lycophyte and monilophyte clades form a grade with respect to a monophyletic clade containing all seed plant SMXLs (Fig. 7). We believe the data are most consistent with there being a complement of 1 *SMXL* gene in the last common ancestor of land plants, and with this basic complement being maintained during much of land plant evolution (Fig. 7).

In gymnosperms, there is one clade in each of the *SMAX1* and *SMXL4* super-clades, which we denote as *gSMAX1* and *gSMXL4* (Table 2). Our analysis suggests that there was a duplication in the *SMAX1* and *SMXL4* super-clades at the base of angiosperms, such that all angiosperms have at least four *SMXL* genes: *SMAX1* and *SMXL78* (*SMAX1* super-clade) and *SMXL39* and *SMXL4* (*SMXL4* super-clade) (Fig. 7). Further duplications at the base of the eudicots has given rise to further sub-clades, *SMXL7* and *SMXL8* (within the *SMXL78* clade) and *SMXL3* and *SMXL9* (within the *SMXL39* clade), such that the basal complement of eudicots is 6 SMXL proteins (Fig. 7). As previously described, *Arabidopsis* has 8 *SMXL* genes [31]: *SMXL3*, *SMXL8* and the pairs *SMAX1*-*SMXL2*, *SMXL6*-*SMXL7* and *SMXL4*-*SMXL5* which all represent recent duplications in the Brassicaceae; *SMXL9* has been lost from the lineage (Fig. 7).

### Diversification of SMXL protein structure in angiosperms

In gymnosperms, there are two unambiguous clades of SMXL proteins, but there are few obvious differences in protein sequence or structure to distinguish the gSMAX1 and gSMXL4 proteins from each other. Indeed, when we examined % amino acid identity across conserved domains, both gSMAX1 and gSMXL4 proteins had ~ 50% identity with non-seed plant SMXL proteins (Table 3), suggesting the proteins conserve the structure of the ancestral SMXL proteins equally well. This tentatively suggests that gSMAX1 and gSMXL4 may simply be sub-functionalized with respect to the ancestral proteins.

**Table 3:** SMXL protein identity comparison

	SMXLA-J	gSMXL4	gSMAX1
gSMXL4	49.9		
aSMXL4	40.0	50.9	
SMXL39	39.9	48.2	
SMXL3	41.6	50.7	
SMXL9	38.6	47.1	
gSMAX1	49.1		
aSMAX1	44.6		60.4
SMXL78	34.3		41.0
SMXL7	34.7		41.2
SMXL8	34.1		40.9

Protein identity comparisons between different SMXL groups. Pairwise identity scores were calculated for each of 167 full-length/near full-length proteins in our alignment, across the 477 amino acids used for phylogenetic reconstruction. For each seed plant sequence, we then averaged their % identity across non-seed plant sequences from SMXLA, SMXLB, SMXLD, SMXLE, SMXLF and SMXLJ clades. We then averaged these scores to reach an average % identity per clade (2nd column). Additionally, for each angiosperm SMXL4/SMXL39/SMXL3/SMXL9 sequence, we calculated % identity with gymnosperm SMXL4 sequences, then averaged across the clade (3rd column). Finally, for each angiosperm SMAX1/SMXL78/SMXL7/SMXL8 sequence, we calculated % identity with gymnosperm SMAX1 sequences, then averaged across the clade (4th column)

Conversely, angiosperm SMXL proteins are much more differentiated from each other. Assuming a parsimonious reconstruction of SMXL evolution (Fig. 7), the aSMAX1 and SMXL78 clades of proteins are equally related to the gSMAX1 clade of proteins from a phylogenetic perspective, but this is not reflected at the protein level. The aSMAX1 proteins closely resemble gSMAX1 proteins, with approximately 60% protein identity across conserved domains (Table 3). However, across the same domains, SMXL78 proteins only shares 41% identity with gSMAX1 proteins (Table 3). Given the well-defined functional data for aSMAX1 and SMXL78 proteins, these data suggest that SMXL78 proteins are likely to be neo-functional and that aSMAX1 retains the ancestral function also present in gSMAX1 proteins. However, the data are not conclusive, and it is possible that gSMAX1 proteins perform the roles of both aSMAX1 and SMXL78 and that aSMAX1 and SMXL78 are sub-functionalized relative to their common ancestor.

More dramatic are the changes in protein structure seen in the aSMXL4, SMXL3 and SMXL9 clades. In *Arabidopsis*, SMXL3, SMXL4 and SMXL5 have been characterized as non-labile, on the basis that they lack the FRGKT motif that confers proteolytic lability on SMXL7, and their observed increased stability relative to SMAX1 [39]. This FRGKT motif is broadly present in all non-seed plant SMXLs, and in SMAX1 and SMXL7/SMXL8 proteins, but is absent from all SMXL3, SMXL4 or SMXL9 proteins in angiosperms (Additional file 14). Conversely, we observed that SMXL4 proteins from gymnosperms still retain the FRGKT motif. This suggests that non-lability is an angiosperm-specific innovation that occurred after the angiosperm-gymnosperm split, but before the duplication that led to the separation of the aSMXL4 and SMXL39 clades. While the affinity of aSMXL4 proteins to gSMXL4 proteins is not as strong as that observed between aSMAX1 and gSMAX1, there is still 51% identity across the conserved domains of these proteins, suggesting that aSMXL4 could still retain the same function as gSMXL4, albeit without the FRGKT motif (Table 3).

In addition to lacking the FRGKT motif, SMXL3 and SMXL9 also completely lack part of the second NTPase domain at the C-terminus of the protein, which has recently been suggested to mediate interaction with D14 in SMXL7 [11]. This domain is likely to broadly mediate interactions between SMXL proteins and members of the D14/KAI2/DLK2 family, and the reduction of this domain in SMXL3 and SMXL9 in turn suggests that the activity of these proteins is not regulated by D14/KAI2 proteins. In contrast, aSMXL4 proteins have intact second NTPase domains, suggesting they do interact with D14/KAI2 family members, albeit in a way that does not alter their stability.

## Discussion

### Ancient enzymes, ancient signals

**Fig. 8:**
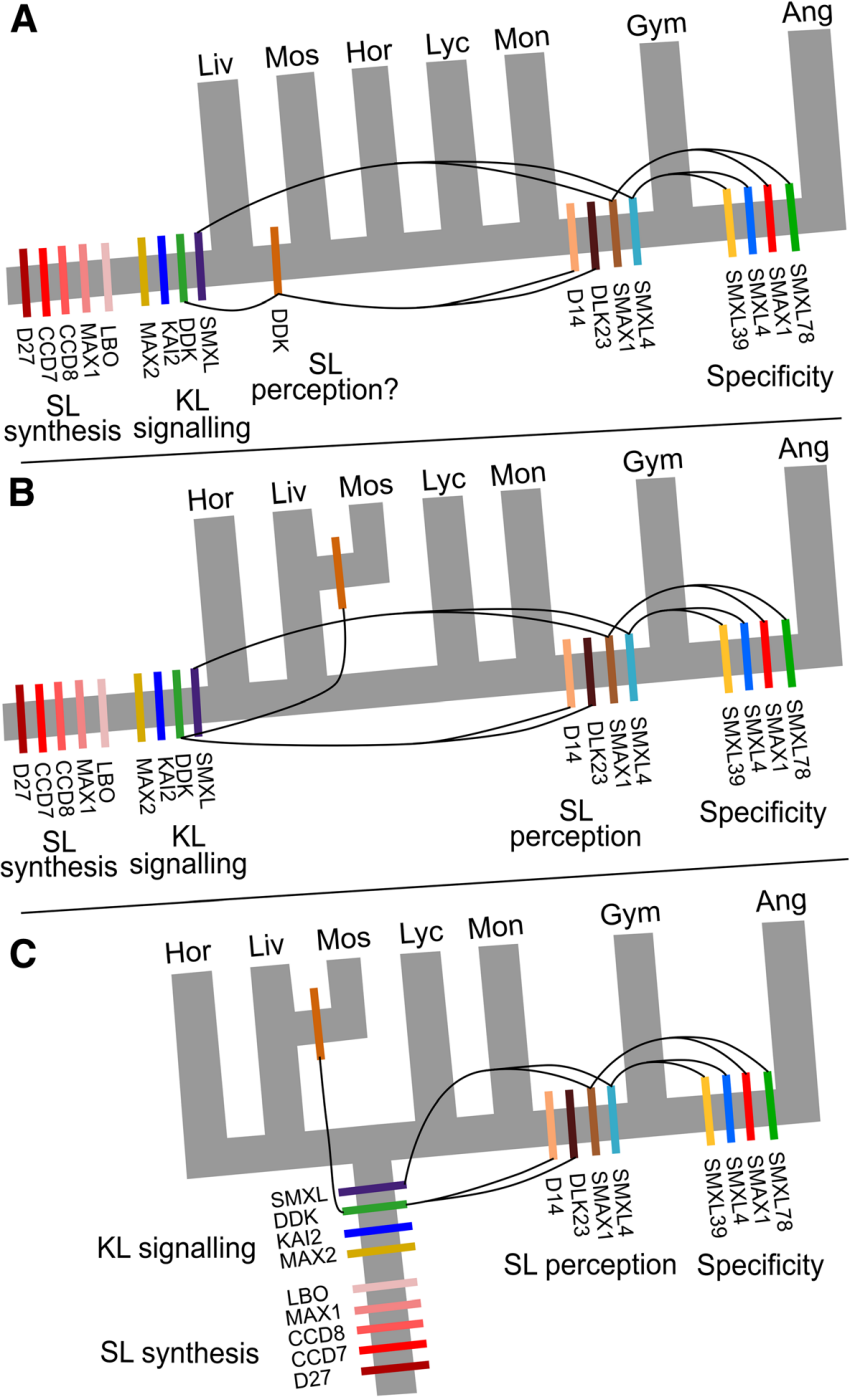
Models of strigolactone signalling evolution. Three models of land plant evolution, showing the likely origins of different protein families involved in SL/KL synthesis and signalling. **a** Traditional view [37]. **b** ‘Hornworts-basal’ model [38]. **c** ‘Monophyletic bryophytes’ [44]. Depending on the scheme of evolution, the likely origin(s) of SL perception vary considerably. Curved lines represent the innovations (duplications, sub/neo-functionalization) in the DDK and SMXL lineages implied by each scenario. Hor = hornworts, Liv = liverworts, Mos = mosses, Lyc = lycophytes, Mon = monilophytes, Gym = gymnosperms, Ang = angiosperms

A large and growing number of SL-type molecules have now been identified from root exudates [28]. Synthesis of SLs has been reported from across the land plant clade, including in liverworts [24], mosses [26], lycophytes, gymnosperms and angiosperms [28]. This apparent broad distribution has been paradoxical when set against the apparent absence of core SL synthesis enzymes in many model species; for instance, *Marchantia polymorpha* lacks a *CCD8* and *MAX1* orthologue, while *Physcomitrella patens* also lacks *MAX1* (reviewed in [1]). This has led to the suggestion of non-canonical synthesis pathways for SLs [1, 40]. However, there appears to be a rather more straightforward answer to this paradox. Our re-examination demonstrates that the complete set of core SL synthesis enzymes, probably including the recently identified LBO oxidoreductase, are present across land plants (Fig. 8). Clearly, as with *M. polymorpha* and *P. patens*, individual species have lost genes, but taken as a group, we found all the core enzymes present in liverworts, mosses, lycophytes, gymnosperms and angiosperms. From hornwort transcriptomes, we only identified *CCD8* and proto-*LBO* sequences, but this may be due to relatively low number of transcriptome assemblies in this taxon, especially if the tissues expressing SL synthesis genes were not used to generate the transcriptomes. However, we cannot exclude the alternative possibility that hornworts have lost *D27*, *CCD7* and *MAX1* genes, and may not synthesize strigolactones at all. It is notable that across all taxa, we struggled to identify *D27* and *CCD7* sequences from transcriptome assemblies, even when unambiguously present in the fully sequenced genomes of related species. This suggests these genes have very low or very spatially restricted expression across land plant taxa. While the predecessors of these SL synthesis enzymes are present in charophyte algae, specific SL synthesis enzymes do not seem to be present in charophytes [24, 41]. A case has been made for the presence of D27 enzymes in charophyte algae [24], but we suggest that these proteins are actually more likely to represent D27-LIKE1 enzymes, with D27 forming a land-plant-specific innovation (Fig. 1).

Overall, the evolutionary distribution of SL synthesis enzymes is broadly consistent with the reported detection of SLs themselves. However, some caution is required, as it now appears that early mass spectrometry experiments have produced false-positive signals for SLs in several species (reviewed in [28]). Thus, while *P. patens* was previously found to produce several SLs, including some even in the absence of CCD8 [26], re-examination of this species failed to identify any SLs other than carlactone [28]. This is consistent with the presence of D27, CCD7 and CCD8 in *P. patens* and the lack of MAX1. In a similar vein, *M. polymorpha* was previously reported to produce SLs [24], but this seems unlikely given the lack of CCD8 and MAX1 in this species, although we cannot exclude the possibility of non-canonical synthesis pathway. Perhaps most pertinently, it was previously suggested that charophyte algae in the Charales produced the derived SL sorgolactone [24]. Given the absence of true CCD7 and CCD8 enzymes in charophyte algae, the failure to detect SLs in other charophyte algae [24] and the acknowledged issues with the false-positive detection of SLs in past studies [28], this tentatively suggests that the detection of sorgolactone in these species could have been a false positive. We hypothesize that SLs are only produced in land plants, consistent with the apparent evolution of true SL synthesis enzymes at the base of land plants (Fig. 8).

### A late origin for strigolactone signalling?

Since mosses and angiosperms both display developmental responses to SLs, the tendency has been to assume that SL perception—of some form—evolved early in land plant evolution (e.g. [42]). Our previous work showed that the evolution of eu-D14 type SL receptors only occurred relatively recently in the seed plants [29]. However, it has been demonstrated that in the ‘DDK’ sub-family of the D14/KAI2 family, there are proteins in mosses, lycophytes and monilophytes that could potentially act as SL receptors [29, 43]. Conversely, in liverworts, proteins in the DDK sub-family are unambiguously KAI2-like [29]. On this basis, and given a traditional model of land plant phylogeny, we proposed an ‘early’ origin for SL signalling, after the divergence of liverworts and all other land plants [29] (Fig. 8a). However, the data we present in the current study calls this idea into question. For instance, we provide evidence for canonical strigolactone synthesis in liverworts, species that have no obvious mechanism for strigolactone perception. We also show that the origin of specific strigolactone-targeted SMXL proteins occurs very late in land plant evolution, inconsistent with the idea of an early origin for canonical strigolactone signalling.

The interpretation of the available evidence must also take into account major developments in our understanding of land plant evolution. A recent study has discounted the traditional view of land plant evolution in favour of a ‘monophyletic bryophyte’ model (Fig. 8c), with the previously controversial ‘hornworts-basal’ model also remaining plausible (Fig. 8b) [44]; in both models, liverworts and mosses are sister taxa. Given that there is no evidence for canonical SL signalling in liverworts, then if liverworts and mosses are sister taxa, any DDK-based strigolactone SL in mosses must have evolved independently from D14-mediated SL signalling in seed plants. Indeed, there is currently a lack of unambiguous evidence for any form of SL perception in liverworts, hornworts, lycophytes and monilophytes. While developmental responses to SL treatment have been suggested in *Chara corallina* and liverworts [24], these assays all used *rac*-GR24, and thus, the responses cannot confidently be attributed to SL-like molecules. Given the currently favoured models of land plant evolution, the known distribution of developmental SL perception among land plants and the phylogenetic distribution of D14 and SMXL7/D53 proteins, our data support a late, rather than early, origin for canonical SL signalling specifically in angiosperms, with an independent origin of SL perception in mosses (Fig. 8b, c).

If mosses have convergently evolved SL perception, the question remains as to whether this occurred through independent recruitment of DDK proteins. Currently, the balance of evidence is slightly against this idea. There are some DDK proteins from *Physcomitrella patens* that may have D14-like binding pockets [43], and although this is not a general feature of moss DDK proteins [29], there is no particular reason for them to directly resemble D14 if they have evolved convergently. However, where tested, these *P. patens* proteins do not bind strigolactones [45]. The *P. patens* MAX2 protein does not obviously act in SL responses [46], but the moss DDK proteins lack some of the key MAX2-interaction residues [29], so if they are SL receptors, this would be consistent with MAX2-independent action. The emergence of the second SMXLC lineage in Bryopsidan mosses is also intriguing; could this represent the convergent evolution of specific SL-targeted SMXL proteins? More work is certainly warranted to investigate the role of DDK and SMXL proteins in SL perception in mosses.

If SLs are not developmental regulators in most non-seed plants, then why are they (apparently) synthesized in these species? The most obvious answer would be that SLs evolved firstly as rhizosphere signalling molecules and would have subsequently been recruited as ‘internal’ hormones in seed plants by evolution of SL signalling. Indeed, before strigolactones were identified as hormones in flowering plants, it has been proposed that SLs are ancestral rhizosphere signals [47]. In this respect, it is worth considering the case of *M. polymorpha*, which has lost SL synthesis enzymes relative to its close relatives *M. paleacea* and *M. emarginata* (Additional file 8, Additional file 10), and has also lost the ability to form mycorrhizal symbioses relative to these species. While it is difficult to disentangle cause and effect here, this is unlikely to be coincidental. Mosses as a group have lost the ability to form mycorrhizal associations, and this might be connected to their independent ability to use SL in the regulation of development. The main role of SLs in *P. patens* seems to be in the regulation of colony morphology and ‘quorum sensing’ [26], and this can be conceptualized as an alternative use of SLs as rhizosphere signals, rather than recruitment as true hormones.

### An ancient KAI2-SMXL module

By contrast with SL signalling, there is little uncertainty regarding the origin of KAI2 signalling in land plants, since KAI2 proteins are present across the clade and proto-KAI2 proteins are found in charophyte algae [29]. Here we have shown that SMXL proteins are also found throughout land plants and that in most non-seed plants, only a single SMXL type is found. We also show that these non-seed plant SMXLs have the FRGKT motif found in D53/SMXL7, suggesting that they may be degraded in a SCF^MAX2^-dependent manner. Given the ubiquity of KAI2 proteins, it seems most likely that SMXL proteins in non-seed plants are degraded in response to KAI2 activation, and indeed, we have shown that, of SMXL proteins found in angiosperms, non-seed plant SMXL proteins most closely resemble SMAX1, the presumptive target of KAI2 signalling. Although evidence is only circumstantial at this point, we believe that KAI2-induced degradation of SMXL proteins is an ancestral signalling mechanism in land plants.

**Fig. 9:**
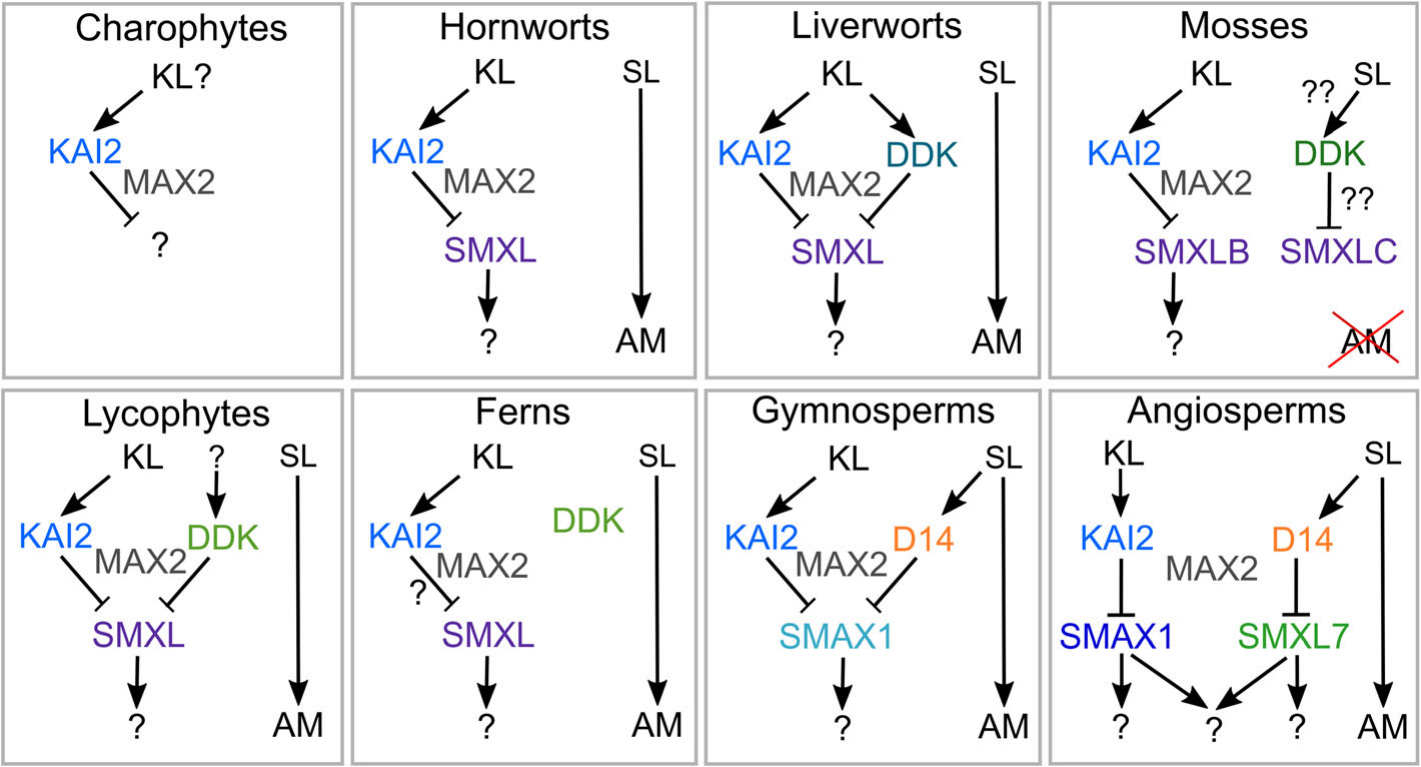
Ligand and target specificity in D14/KAI2 signalling. Possible models of D14/SMXL signalling in different land plant groups, based on (1) identified D14/KAI2 and SMXL proteins in each taxon, (2) presence or absence of MAX2-interaction residues in D14/KAI2 proteins and (3) predicted shape of ligand binding pockets in D14/KAI2 proteins [29]. In charophyte algae, there may be a KAI2-MAX2 signalling module, but there are no apparent SMXL proteins, and the function of this complex is not clear. In hornworts and liverworts, there is likely to be canonical KAI2 signalling, but there does not seem to be any canonical SL signalling, and SLs may act only as mycorrhizal signals. In mosses, mycorrhizal associations have been lost, and SL may have been independently recruited as a hormonal or ‘quorum sensing’ signal. This may plausibly have occurred by neo-functionalization of DDK and SMXL proteins, but either way is MAX2 independent. In both lycophytes and monilophytes, the status of SL signalling, and whether there is any connection with DDK proteins, is largely unclear. In monilophytes, DDK proteins probably act independently of MAX2, and it is unclear with SMXL proteins interact with the KAI2-MAX2 module. In gymnosperms, canonical D14-mediated SL signalling is present, but both SL and KL probably target the same SMXL protein for degradation. In angiosperms, SL and KL target separate SMXL proteins for degradation. It is unclear whether the downstream targets of the pathways are still shared, or have also separated

We previously proposed that canonical SL signalling arose from neo-functionalization of KAI2-family receptors in seed plants, a process which was certainly complete by the last common ancestor of extant seed plants [29]. However, gymnosperms only have two classes of SMXL protein, neither of which resembles SMXL7/D53 proteins, and our phylogenetic analysis clearly shows *SMXL7* arising from within the *SMAX1* lineage in angiosperms. Thus, while SL and KL signalling have different target proteins in angiosperms, our analysis suggests they target the same protein for degradation in gymnosperms and thus presumably regulate the same downstream developmental processes (Fig. 9). This raises the question as to whether KL and SL signalling also essentially regulate the same downstream processes in angiosperms or whether the SL signalling pathway in angiosperms has evolved completely novel targets relative to KL signalling (Fig. 9).

It thus seems likely that in seed plants there is ancient KAI2-SMXL/SMAX1 module for KL signalling, coupled with a modern D14-SMXL7 module for SL signalling. What about in other land plant lineages? As discussed above, with the possible exception of mosses, there is no clear evidence that non-seed plant DDK proteins act as SL receptors, but the available structural evidence suggests that these DDK proteins interact with MAX2 [29]. Thus, if KAI2-MAX2 interactions in these species do indeed lead to degradation of SMXL proteins, it should also be expected that DDK-MAX2 interactions lead to degradation of SMXL proteins. Given that there is only one SMXL protein in most non-seed plant species, this would further imply that KAI2 and DDK proteins in these species have the same target (Fig. 9). Indeed, in liverworts, proteins of the DDK lineage have no major structural distinction from KAI2 proteins and should probably be viewed as sub-functionalized paralogues of KAI2, rather than neo-functionalized proteins [29]. Overall, we hypothesize that DDK-SMXL interactions are present in seed plants, though the nature of the DDK ligands remains an open question.

Against this general pattern, KAI2/DDK signalling in monilophytes poses something of a conundrum. Monilophyte KAI2 proteins have the hallmarks of MAX2 interacting proteins, and monilophyte SMXL proteins contain an apparent FRGKT-type motif. However, monilophyte DDK proteins lack the MAX2 interaction interface [29], and monilophyte SMXL proteins lack part of the C-terminus of the protein, which in D53 is proposed to be involved to binding to D14 [11]. It is thus likely that KAI2 (but not DDK) and MAX2 interact in monilophytes and that SMXL proteins might be proteolytically degraded, but the available evidence suggests these events may be unrelated, or may occur through a considerably altered interaction topology (Fig. 9).

Thus, across the land plant group, there remain many unanswered questions about the nature and origin of the KL and SL signalling pathways, and their interconnection with each other. Unlike many signalling pathways, the KAI2/DDK-SMXL signalling systems appear to be evolutionarily labile across land plant evolution, and in each major lineage, they are used differently (Fig. 9). They thus form a very interesting system to study evolution of hormonal signalling, and further investigation across different land plant groups is highly warranted.

## Materials and methods

### Bioinformatic retrieval of SL synthesis/signalling sequences

Members of the *D27*, *CCD7*, *CCD8*, *MAX1*, *LBO* and *SMXL* families were initially identified by BLAST searches against complete, annotated genomes from two major sources: Phytozome (www.phytozome.net) or the genome portals for individual species. BLAST searches were performed using the coding sequences from *Arabidopsis thaliana*. Previous analyses of D27, CCD7, CCD8 and MAX1 have shown that members of these protein sub-families are generally highly distinct from other members of the isomerase, carotenoid cleavage dioxygenase and cytochrome P450 families. In the majority of cases, only single sequences are present for these sub-families in each species, and therefore, we only took hits with very low *E* values (1 × 10^−80^ or below). For LBO, where the phylogenetic situation is less clear, we sampled more extensively, taking hits with E values below 1 × 10^−50^. For SMXL proteins, we took any hits with that matched the target sequence across the full length of the protein; these generally had *E* values below 1 × 10^−50^. Reciprocal BLASTs were performed with recovered sequences to confirm that only true homologues of the target gene in question had been identified.

Preliminary alignments and trees were assembled using these complete sequences and were used to guide the iterative interrogation of transcriptome databases, particularly those generated by the 1KP project (https://sites.google.com/a/ualberta.ca/onekp; https://db.cngb.org/onekp). For transcriptome datasets, we used BLASTn to probe each major taxonomic group for any sequences matching the target sequence from *Arabidopsis*. Generally, only closely related sequences were identified through this approach, and no cut-off was used. For non-annotated sequences from transcriptome datasets, we searched translations across all 6 reading frames to identify ORFs, and the longest ORFs were extracted for alignment. Any ORFs that did not match the target sequence were discarded at this point. All sequences identified in this study are listed in Additional file 1.

### Alignment

Alignments were initially performed in BioEdit [48] using ClustalW [49] with default settings. Full-length nucleotide sequences from completed genomes were loaded into BioEdit and toggled to amino acid sequences for alignment, which were manually refined as necessary. Alignments were stored as nucleotide level sequence, allowing us to generate coherent nucleotide and amino acid alignments from the same aligned datasets. We then added sequences from transcriptome databases, many of which are incomplete, but the alignment of full-length sequences provided a scaffold to align these sequences correctly. In order to identify the optimal phylogenetic reconstruction for each gene family, we created sub-sets of the full alignments at the nucleotide and their corresponding translated amino acid versions. Improved alignments for the amino acid datasets were obtained using MAFFT [50] using default settings. All amino acid alignments were checked by eye and manually edited in JalView [51], the final alignments used for the analyses are provided in Additional files 2, 4, 5, 8, 10, 12 and 14 and are listed in Additional file 18.

### Phylogenetic reconstruction

To assess the level of phylogenetic signal in the data, we carried out likelihood mapping on all alignments using TreePuzzle [52]. Each dataset is broken into all possible quartets, and the maximum likelihood for each of the three fully resolved trees for those four sequences is estimated. The three likelihoods for each quartet are represented as a single point on an equilateral triangle. Data with well-resolved phylogeny will have points close to the corners of the triangle, and those with poor phylogenetic signal (e.g. star-like phylogeny) will migrate to the central region. Datasets with > 10% of the signal in the centre-most (unresolved) region are considered unsuitable for phylogeny reconstruction (Additional files 16 and 17). For the nucleotide alignments, phylogenies were reconstructed using the maximum likelihood approach using IQTree v.1.6.1 [53] where the best codon model was calculated and then the best tree search was performed with 100 bootstrap replicates. We chose to run the nucleotide analyses using codon models of evolution as these have been found to be more realistic and have also been preferred in previous studies of the evolution of gene family members of the strigolactone signalling [29]. In the case of SMXL, we used RAxML v8.2.9 [54] using the GTR+Gamma model partitioning by the 1st, 2nd and 3rd positions to account for heterogeneity in the sequence. A summary of the best models found per dataset are reported in Additional file 19.

For the amino acid data, the best fitting substitution model was selected for each amino acid alignment using ModelGenerator [55]. Chosen models are reported in Additional file 19. Phylogenetic reconstruction for all amino acid datasets was performed using Bayesian and maximum likelihood methods described below. Bayesian inference analyses were carried out in PhyloBayes v.4.1c [56] using a combination of the CAT model + the best model chosen from ModelGenerator [55] for each dataset (models used reported Additional file 19). We ran two chains for each dataset until convergence was reached, i.e. when the mean difference between the chains was lower than 0.03 (calculated in ‘bpcomp’) using a sampling frequency of 20. Convergence details for each alignment are summarized in Additional file 19. Maximum likelihood analyses on the amino acid data were performed using RAxMLv8.2.9 [54] with 100 bootstrap replicates under the previously determined best fit model (Additional file 19) for each alignment.

### Hypothesis testing

In order to assess the best resulting trees per gene family, we gathered all the resulting topologies in both amino acid and nucleotide alignments and input these into IQ-tree [53] allowing the software to find the best model for the input alignment. We then calculated the per-site-loglikelihood values in IQ-tree and generated 1000 bootstraps to carry out the Approximately Unbiased (AU) test as described in [57].

### Statistical analyses

Independent statistical analyses were not used in this study. The phylogenetic analyses included inherent statistical tests as part of the software; these analyses were performed as described above.

## Additional files

Additional file 1: List of sequences used in this study. Sequences listed in red were not used for phylogenetic analyses on account of incompleteness. (XLSX 32 kb)

Additional file 2: D27 alignment. Trimmed alignment showing all conserved parts of the gene, not only those residues used for phylogenetic reconstruction. (FAS 33 kb)

Additional file 3: Full D27 phylogenies. See figure legends within. (PDF 702 kb)

Additional file 4: CCD7 alignment. Trimmed alignment showing all conserved parts of the gene, not only those residues used for phylogenetic reconstruction. (FAS 54 kb)

Additional file 5: CCD alignment. Trimmed alignment showing all conserved parts of the gene, not only those residues used for phylogenetic reconstruction. (FAS 115 kb)

Additional file 6: CCD phylogeny. See figure legends within. (PDF 138 kb)

Additional file 7: Full CCD7 phylogenies. See figure legends within. (PDF 417 kb)

Additional file 8: CCD8 alignment. Trimmed alignment showing all conserved parts of the gene, not only those residues used for phylogenetic reconstruction. (FAS 84 kb)

Additional file 9: Full CCD8 phylogenies. See figure legends within. (PDF 506 kb)

Additional file 10: MAX1 alignment. Trimmed alignment showing all conserved parts of the gene, not only those residues used for phylogenetic reconstruction. (FAS 47 kb)

Additional file 11: Full MAX1 phylogenies. See figure legends within. (PDF 439 kb)

Additional file 12: LBO alignment. Trimmed alignment showing all conserved parts of the gene, not only those residues used for phylogenetic reconstruction. (FAS 46 kb)

Additional file 13: Full LBO phylogenies. See figure legends within. (PDF 585 kb)

Additional file 14: SMXL alignment. Trimmed alignment showing all conserved parts of the gene, not only those residues used for phylogenetic reconstruction. (FAS 335 kb)

Additional file 15: Full SMXL nucleotide ML phylogeny. Maximum likelihood (ML) tree under the GTR+GAMMA codon model in IQtree. Topology rooted with the hornwort clade. Bootstrap values are shown at each node of the tree. (SVG 171 kb)

Additional file 16: Full SMXL amino acid Bayesian phylogeny. Bayesian inference tree under the CAT + JTT codon model in PhyloBayes. Topology rooted with the hornwort clade. Bootstrap values are shown at each node of the tree. (SVG 161 kb)

Additional file 17: Full SMXL amino acid ML phylogeny. Maximum likelihood (ML) tree with the amino acid dataset under the PROTCATJTTX model in RAxML. Topology rooted at the hornwort clade. Bootstrap values are shown at each node of the tree. (SVG 171 kb)

Additional file 18: Table describing final alignments used for phylogenetic analysis. (PDF 9 kb)

Additional file 19: Table describing final models used for phylogenetic analysis. (PDF 14 kb)

## Data Availability

All data generated or analysed during this study are included in this published article and its additional files.
