# Supplementary material for: Strigolactone synthesis is ancestral in land plants, but canonical strigolactone signalling is a flowering plant innovation
Source: BMC Biol. 2019 Sep 5;17:70. doi: 10.1186/s12915-019-0689-6 (PMC6728956; doi:10.1186/s12915-019-0689-6)
Supplement: Supplementary file 3 — Full D27 phylogenies. See figure legends within. (PDF 702 kb) [file 12915_2019_689_MOESM3_ESM.pdf]

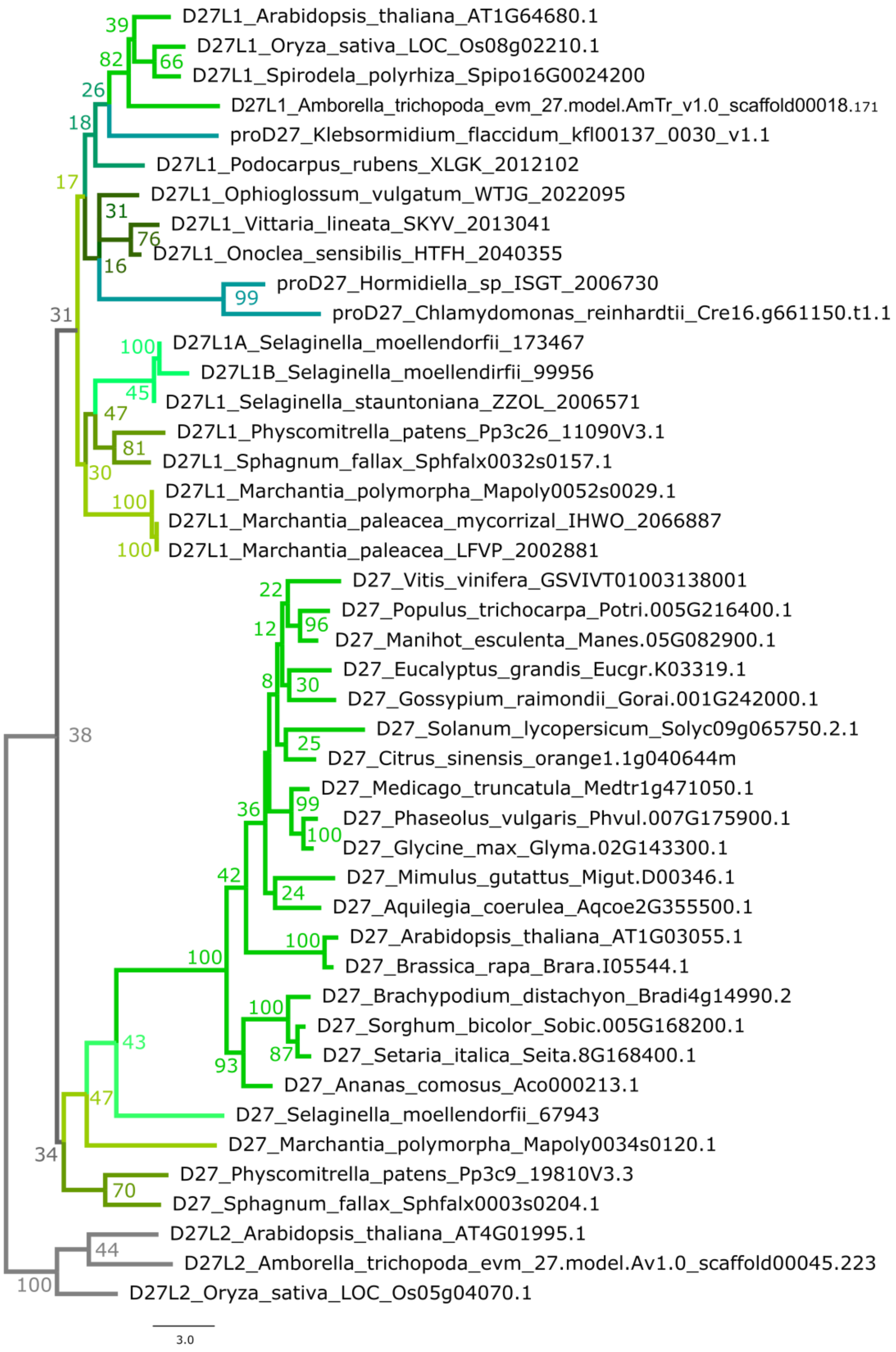

Additional File 3A: Full nucleotide-level ML phylogeny for D27 family

Maximum likelihood (ML) tree under the MGK+F3X4+R5 codon model in IQtree. Topology rooted with the *D27L2* clade. Bootstrap values are shown at each node of the tree.

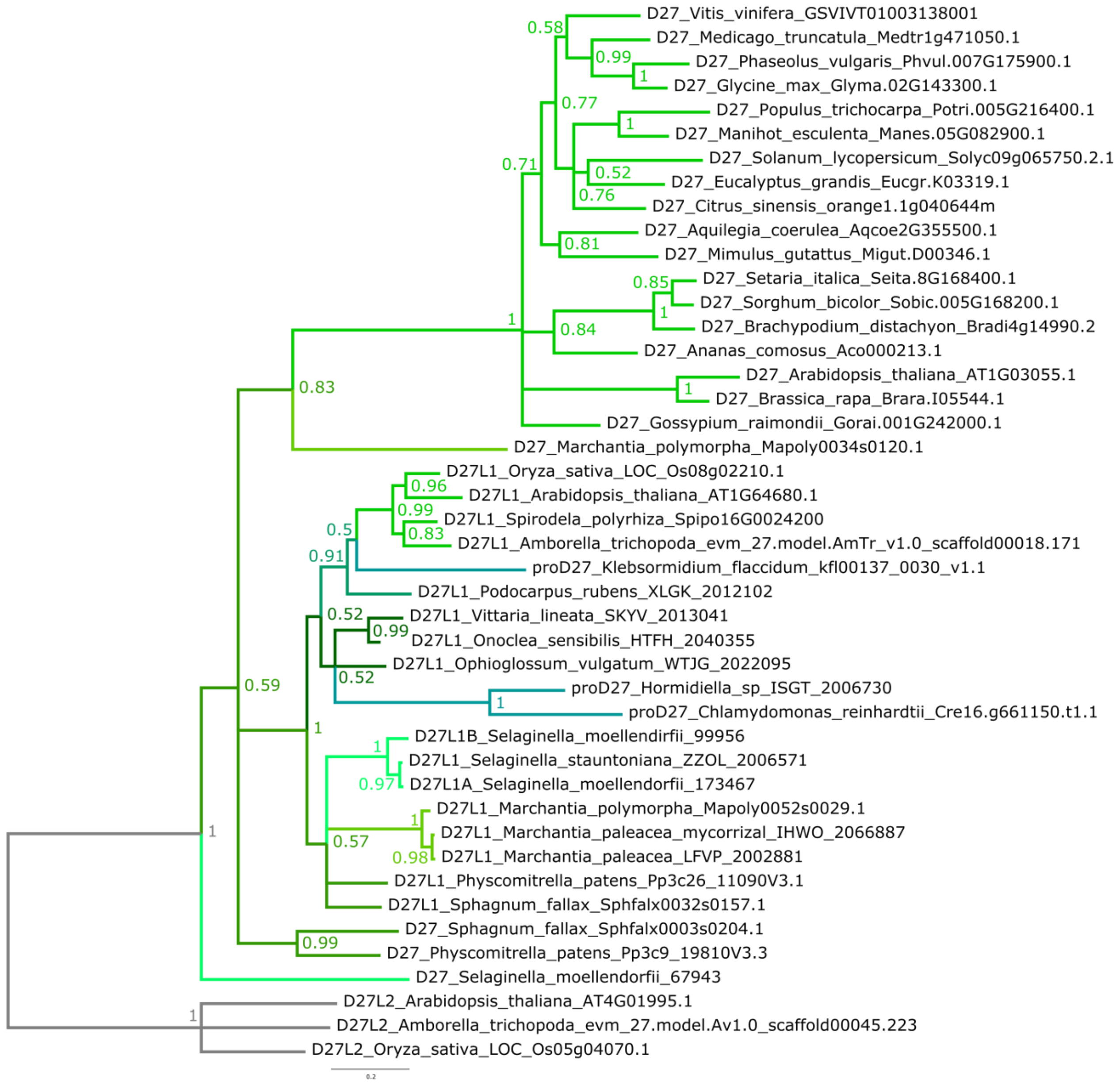

Additional File 3B: Full amino acid-level Bayesian inference phylogeny for D27 family

Bayesian inference tree under the CAT + LG codon model in PhyloBayes. Topology rooted with the *D27L2* clade. Bootstrap values are shown at each node of the tree.

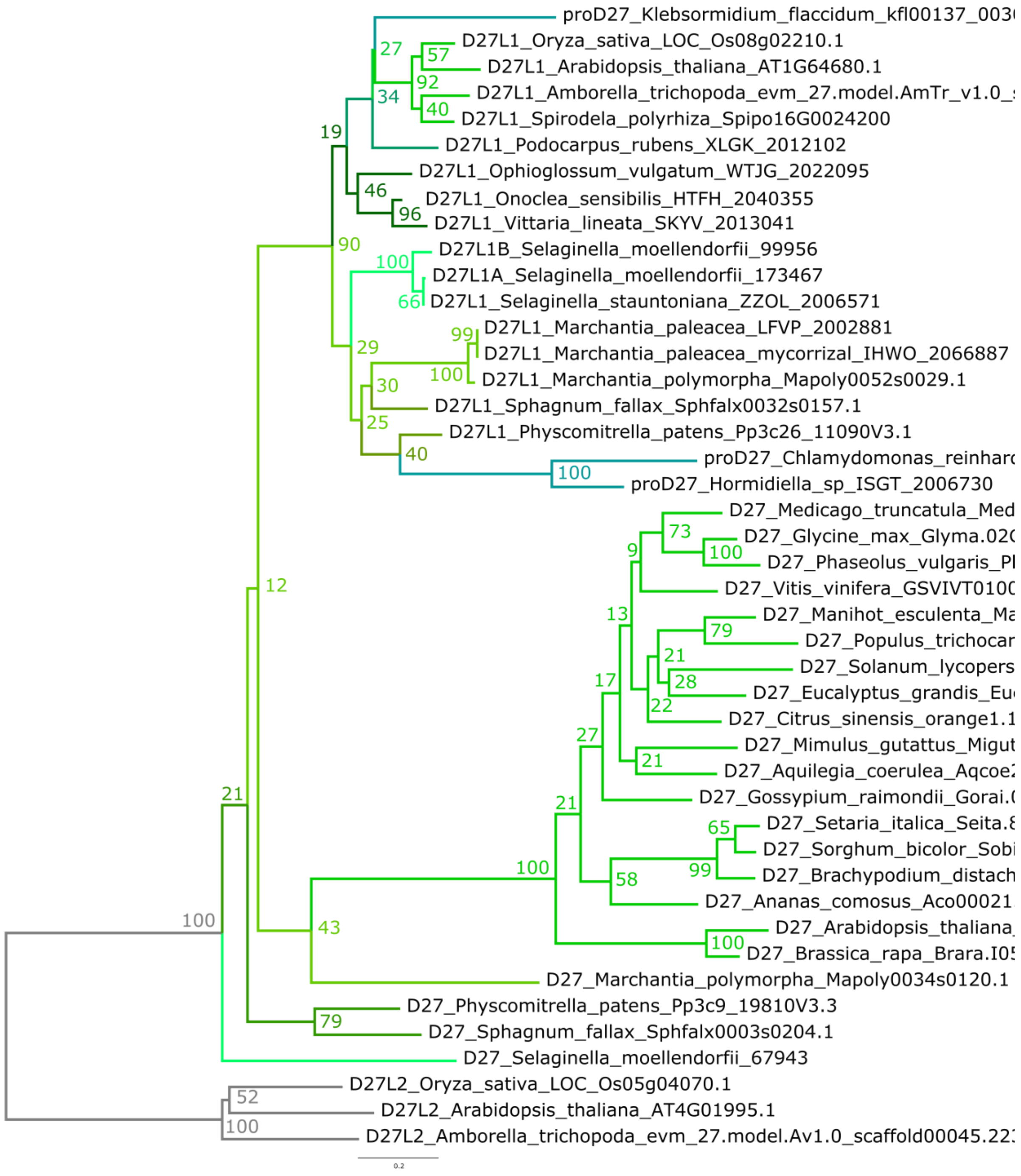

Additional File 3C: Full amino acid-level ML phylogeny for D27 family

Maximum likelihood (ML) tree with the amino acid dataset under the PROTCATLGX model in RAxML. Topology rooted at the D27L2 clade. Bootstrap values are shown at each node of the tree.
