# Supplementary material for: Strigolactone synthesis is ancestral in land plants, but canonical strigolactone signalling is a flowering plant innovation
Source: BMC Biol. 2019 Sep 5;17:70. doi: 10.1186/s12915-019-0689-6 (PMC6728956; doi:10.1186/s12915-019-0689-6)
Supplement: Supplementary file 6 — CCD phylogeny. See figure legends within. (PDF 138 kb) [file 12915_2019_689_MOESM6_ESM.pdf]

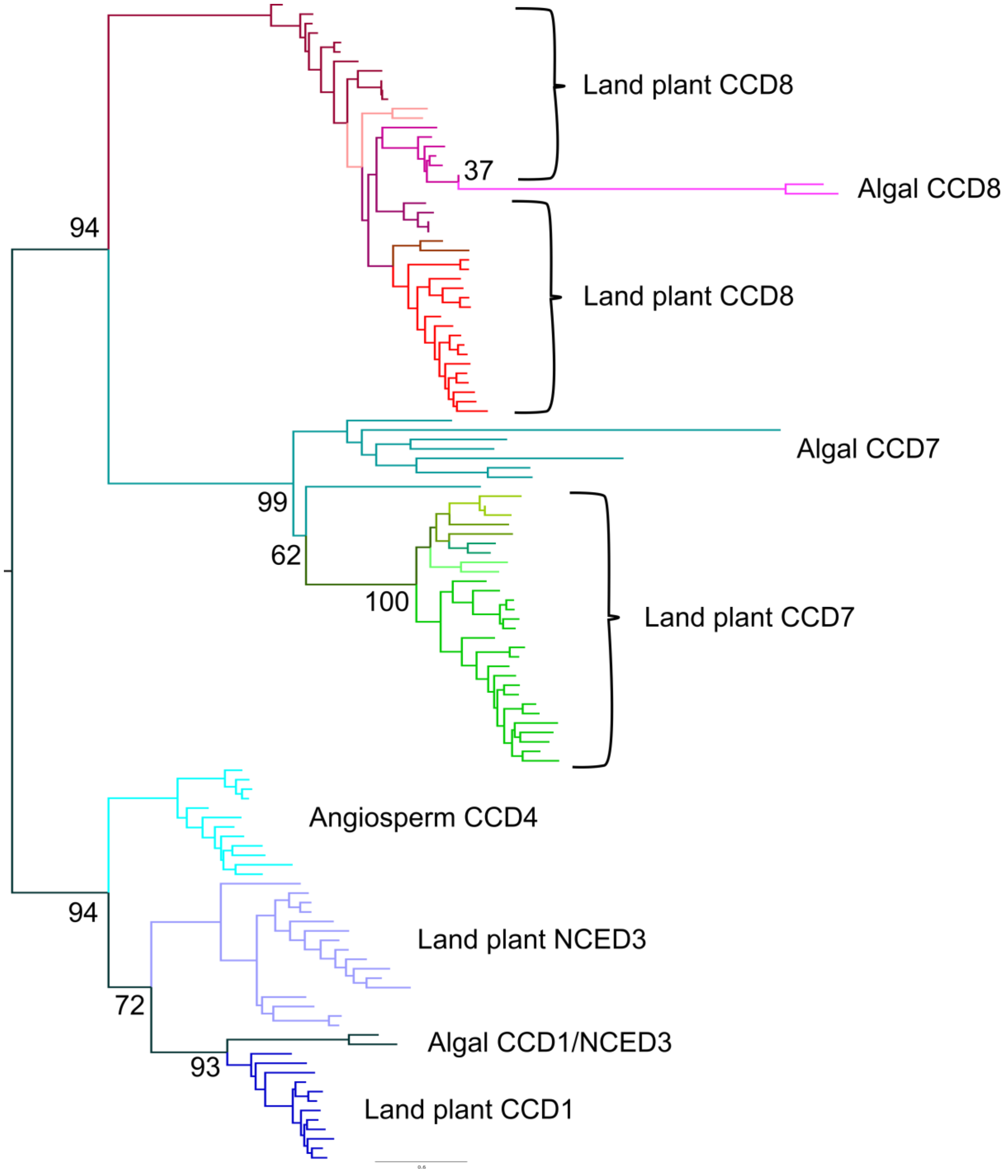

Additional File 6: CCD phylogeny

Nucleotide-level phylogenetic analysis implemented in PhyML on the *CCD* family (123 sequences, 919 characters). The tree was rooted with an algal sequence. Phylogram showing the 'most likely' tree with bootstrap values at key nodes.
