# Supplementary material for: Strigolactone synthesis is ancestral in land plants, but canonical strigolactone signalling is a flowering plant innovation
Source: BMC Biol. 2019 Sep 5;17:70. doi: 10.1186/s12915-019-0689-6 (PMC6728956; doi:10.1186/s12915-019-0689-6)
Supplement: Supplementary file 9 — Full CCD8 phylogenies. See figure legends within. (PDF 506 kb) [file 12915_2019_689_MOESM9_ESM.pdf]

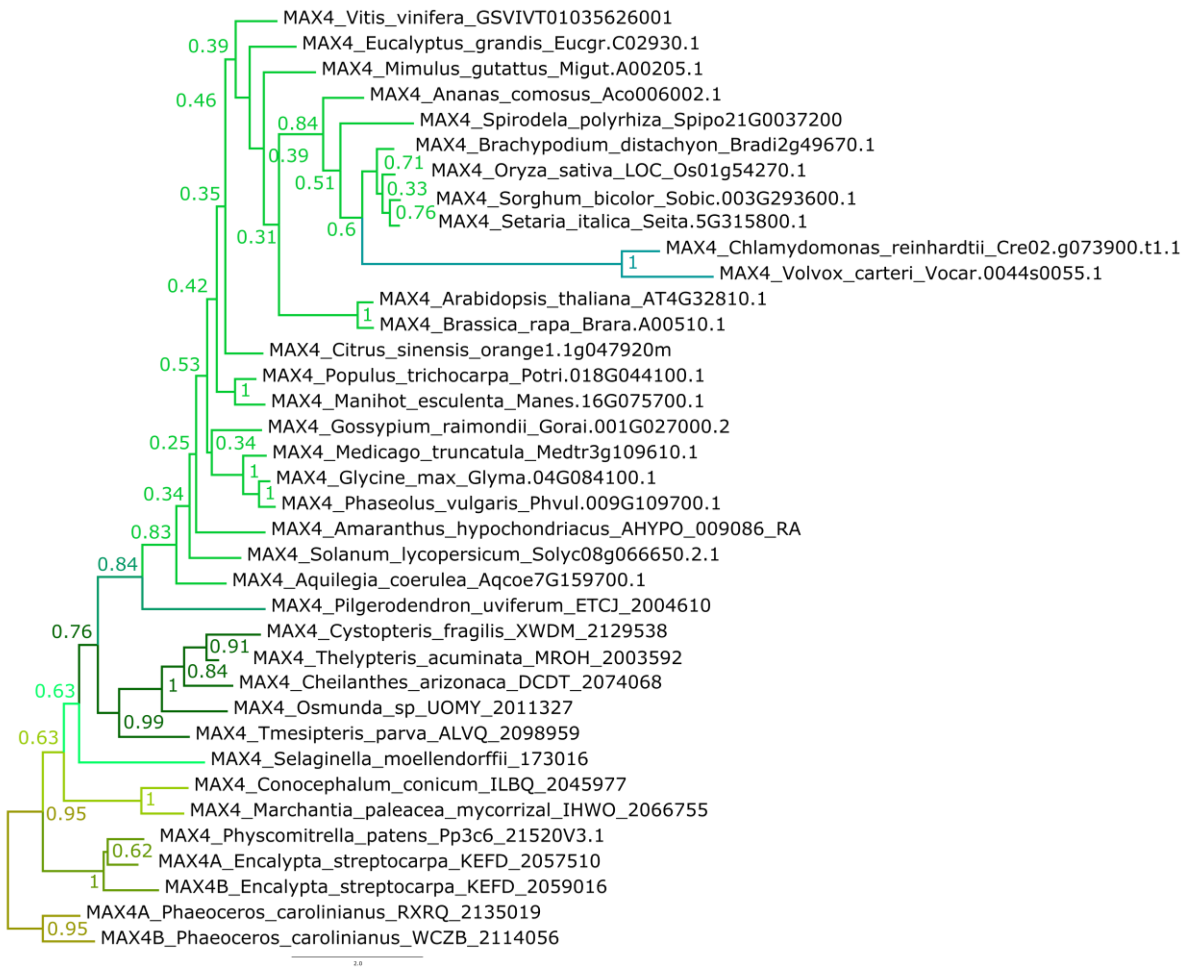

Additional File 9A: Full nucleotide-level ML phylogeny for CCD8 family

Maximum likelihood (ML) tree under the KOSI07+F+R6 codon model in IQtree. Topology rooted with the hornwort clade. Bootstrap values are shown at each node of the tree.

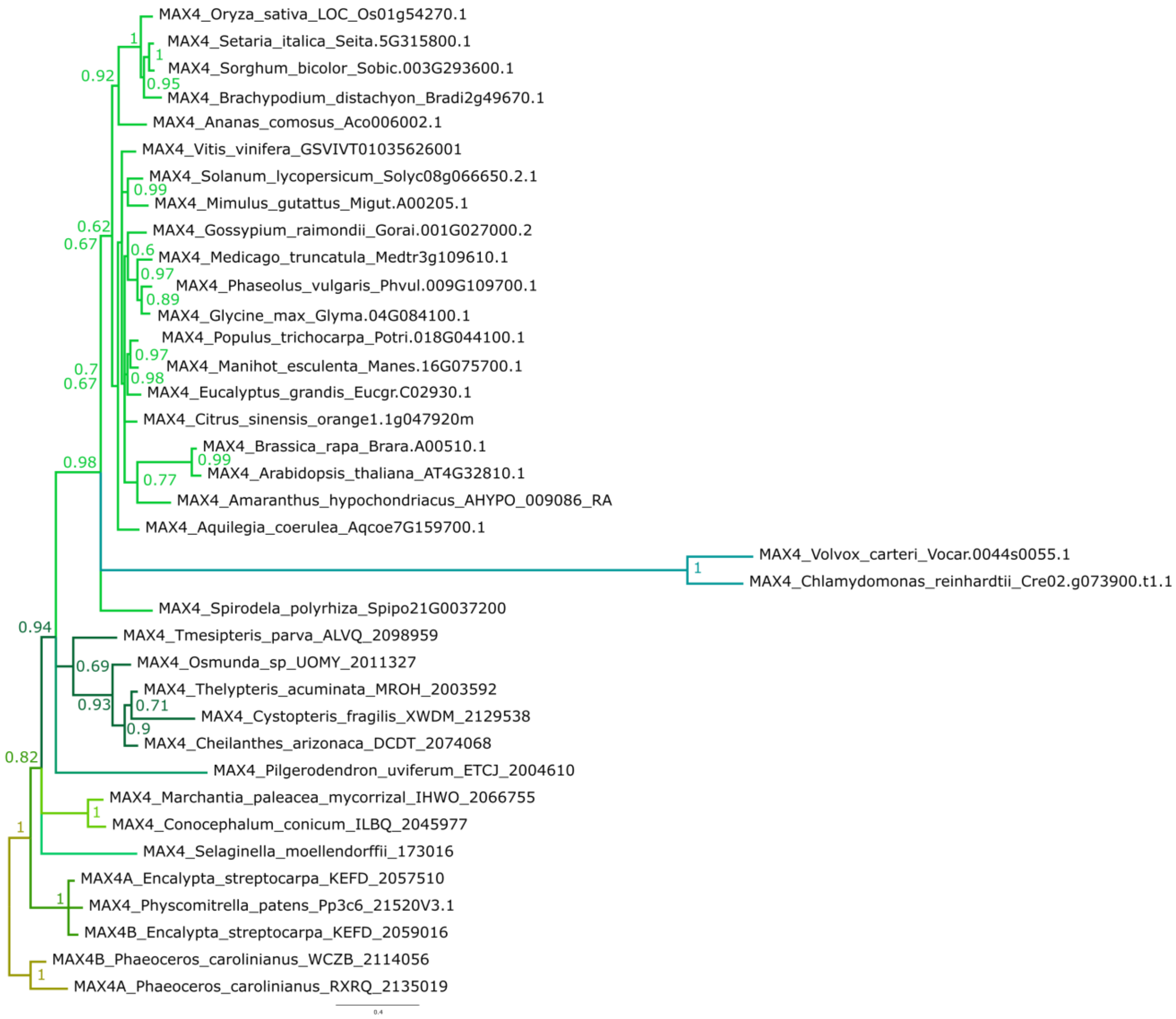

Additional File 9B: Full amino acid-level Bayesian inference phylogeny for CCD8 family

Bayesian inference tree under the CAT + LG codon model in PhyloBayes. Topology rooted with the hornwort clade. Bootstrap values are shown at each node of the tree.

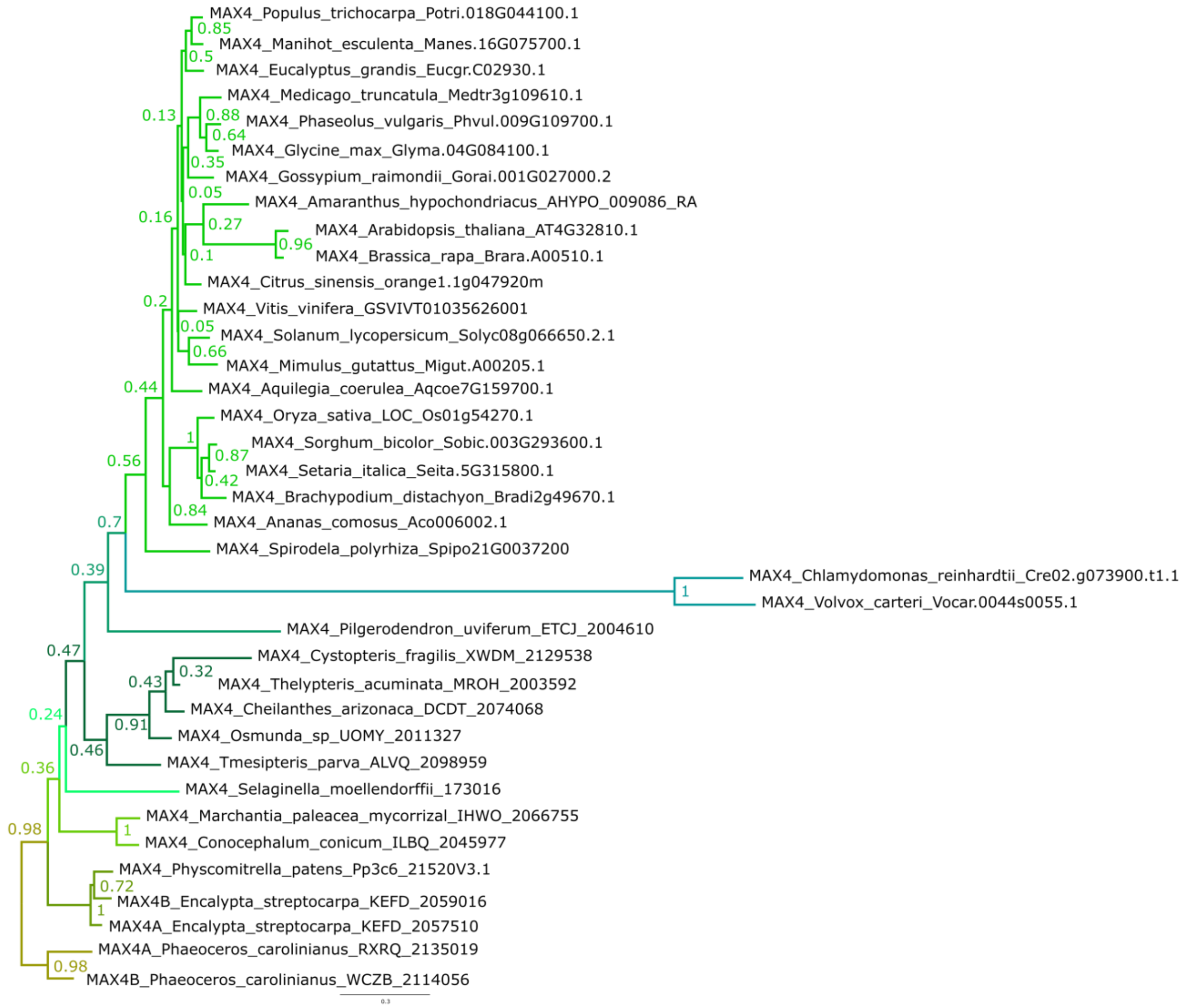

Additional File 9C: Full amino acid-level ML phylogeny for CCD8 family

Maximum likelihood (ML) tree with the amino acid dataset under the PROTCATLGX model in RAxML. Topology rooted at the hornwort clade. Bootstrap values are shown at each node of the tree.
