# Supplementary material for: Strigolactone synthesis is ancestral in land plants, but canonical strigolactone signalling is a flowering plant innovation
Source: BMC Biol. 2019 Sep 5;17:70. doi: 10.1186/s12915-019-0689-6 (PMC6728956; doi:10.1186/s12915-019-0689-6)
Supplement: Supplementary file 11 — Full MAX1 phylogenies. See figure legends within. (PDF 439 kb) [file 12915_2019_689_MOESM11_ESM.pdf]

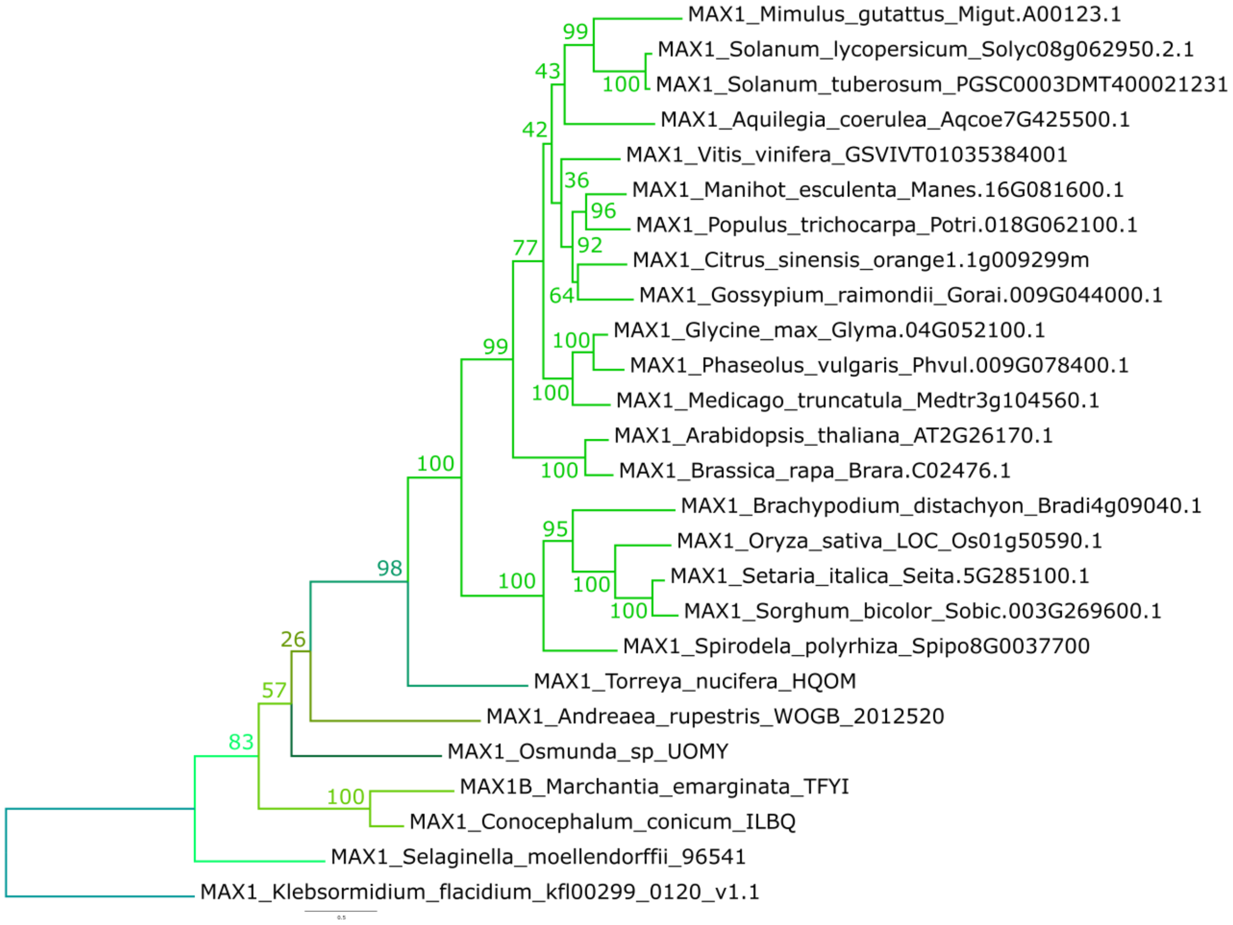

Additional File 11A: Full nucleotide-level ML phylogeny for MAX1 family

Maximum likelihood (ML) tree under the KOSI07+FU+R4 codon model in IQtree. Topology rooted with the algal clade. Bootstrap values are shown at each node of the tree.

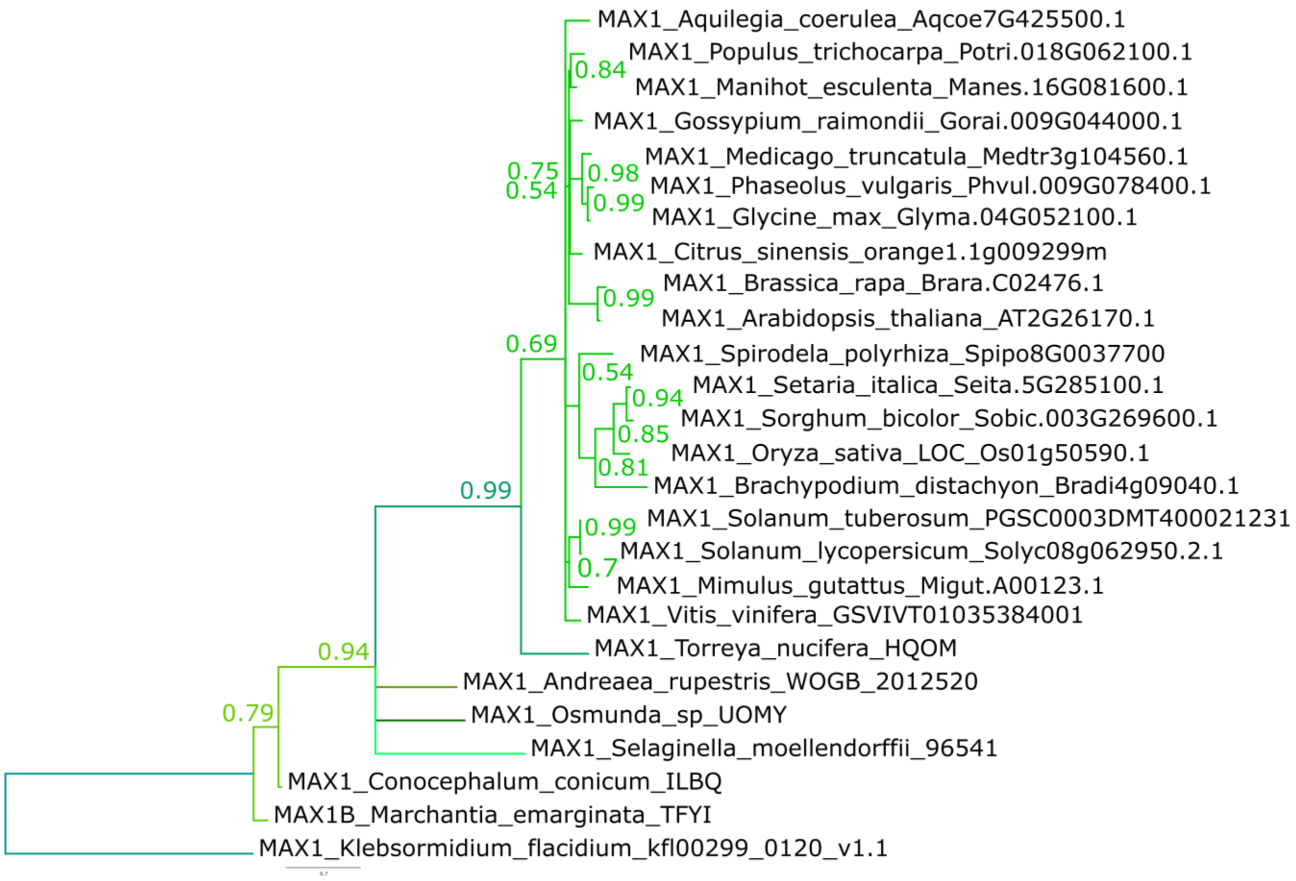

Additional File 11B: Full amino acid-level Bayesian inference phylogeny for MAX1 family

Bayesian inference tree under the CAT + LG codon model in PhyloBayes. Topology rooted with the algal clade. Bootstrap values are shown at each node of the tree.

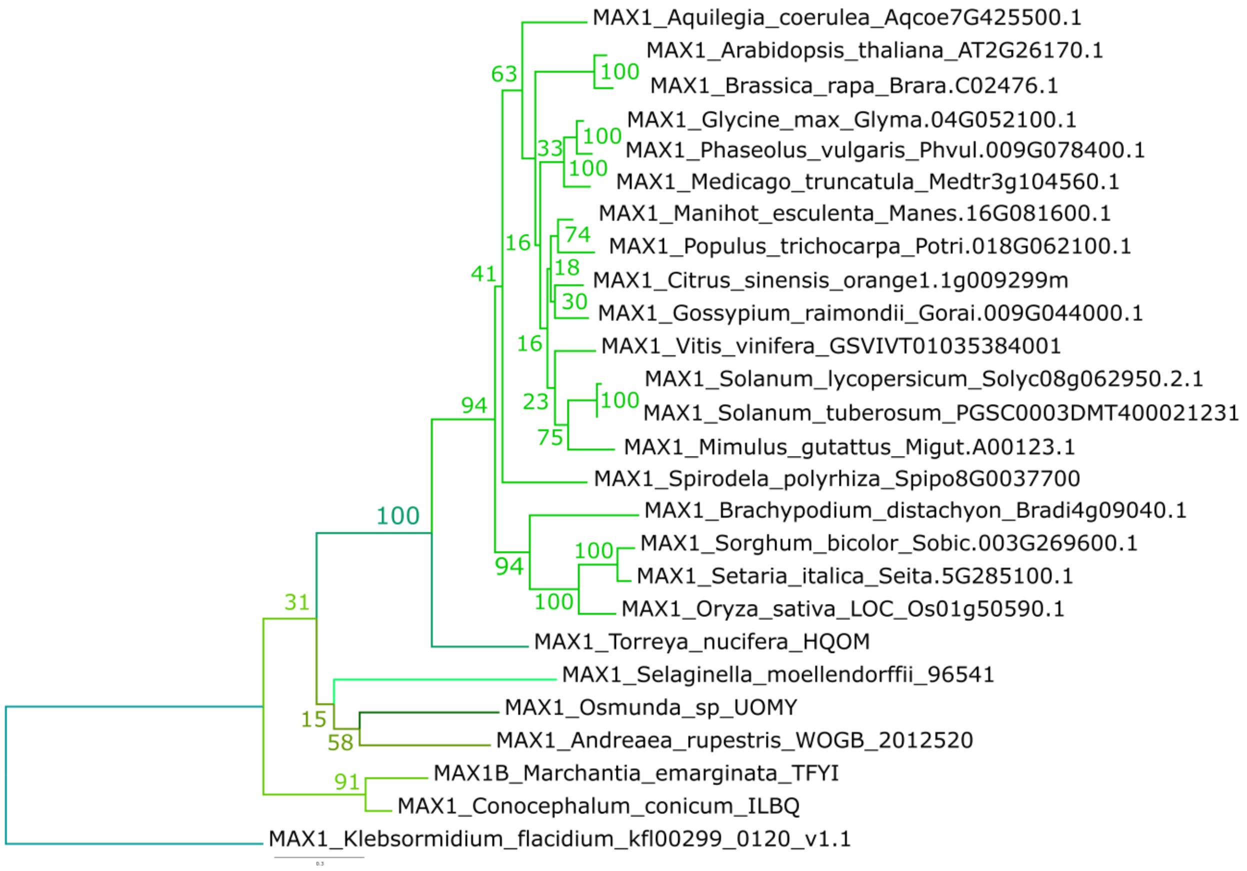

Additional File 11C: Full amino acid-level ML phylogeny for MAX1 family

Maximum likelihood (ML) tree with the amino acid dataset under the PROTCATLGX model in RAxML. Topology rooted at the chlorophyte algal clade. Bootstrap values are shown at each node of the tree.
