# Supplementary material for: Strigolactone synthesis is ancestral in land plants, but canonical strigolactone signalling is a flowering plant innovation
Source: BMC Biol. 2019 Sep 5;17:70. doi: 10.1186/s12915-019-0689-6 (PMC6728956; doi:10.1186/s12915-019-0689-6)
Supplement: Supplementary file 13 — Full LBO phylogenies. See figure legends within. (PDF 585 kb) [file 12915_2019_689_MOESM13_ESM.pdf]

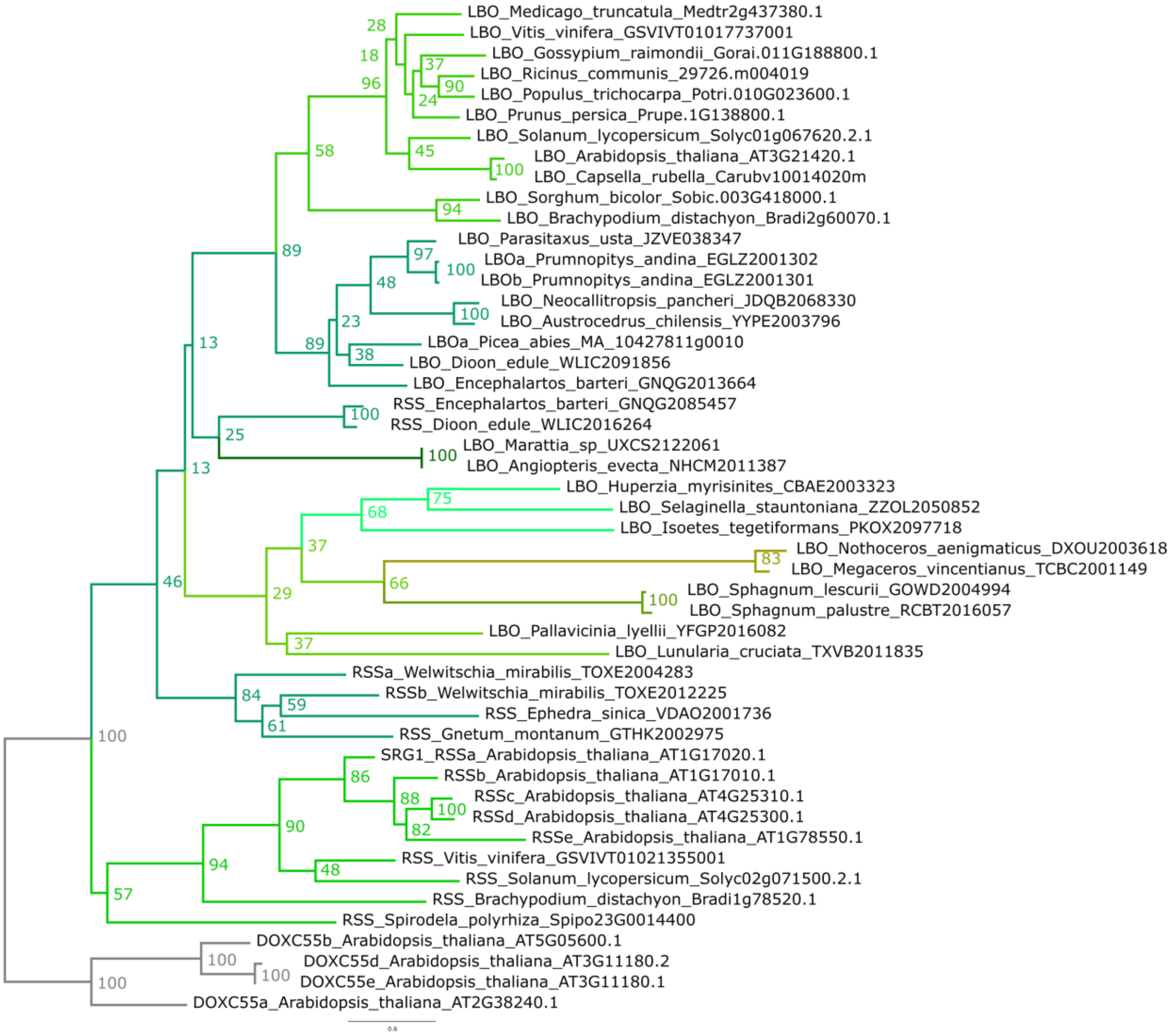

Additional File 13A: Full nucleotide-level ML phylogeny for LBO family

Maximum likelihood (ML) tree under the KOSI07+F+R7 codon model in IQtree. Topology rooted with the DOX55 clade. Bootstrap values are shown at each node of the tree.

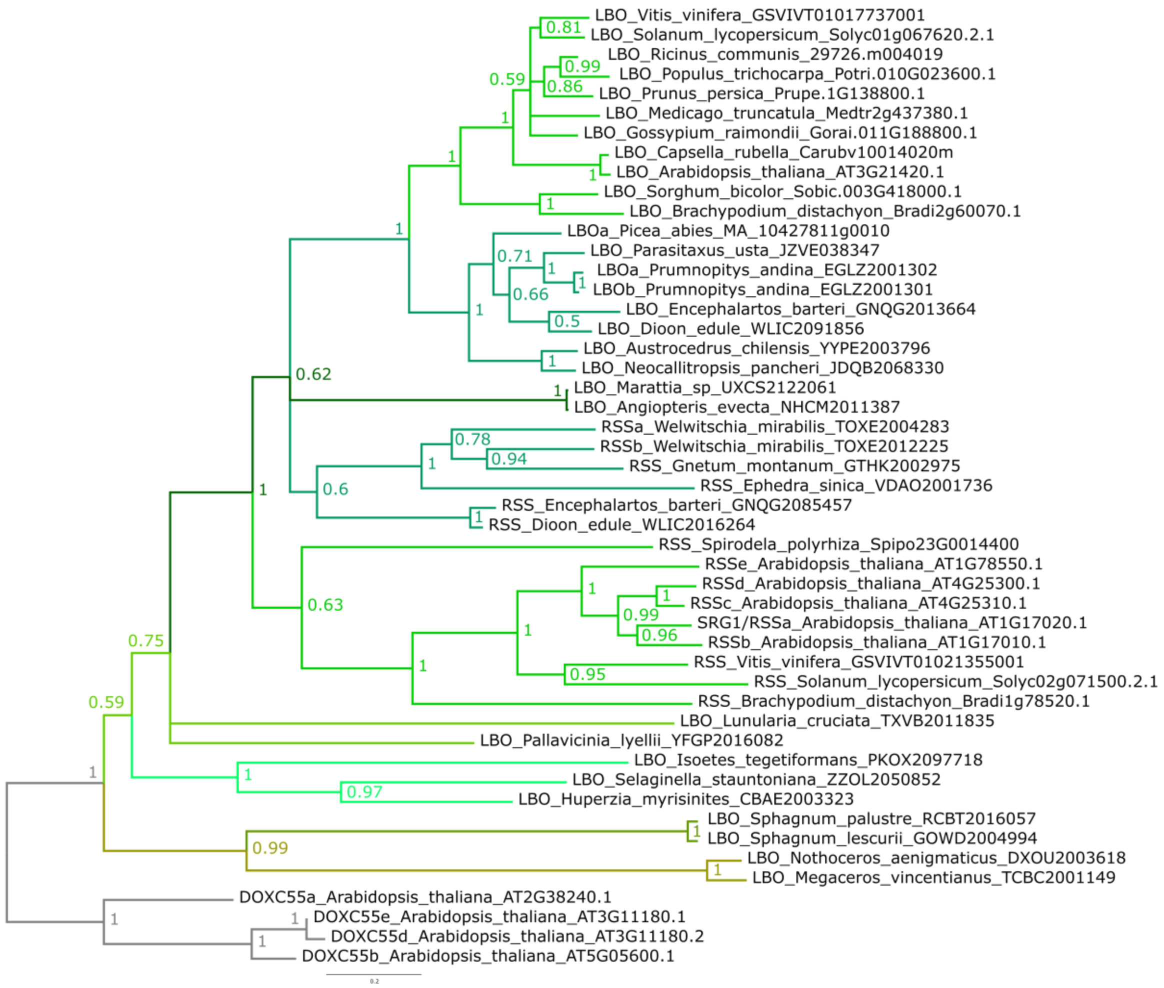

Additional File 13B: Full amino acid-level Bayesian inference phylogeny for LBO family

Bayesian inference tree under the CAT + LG codon model in PhyloBayes. Topology rooted with the DOX55 clade. Bootstrap values are shown at each node of the tree.

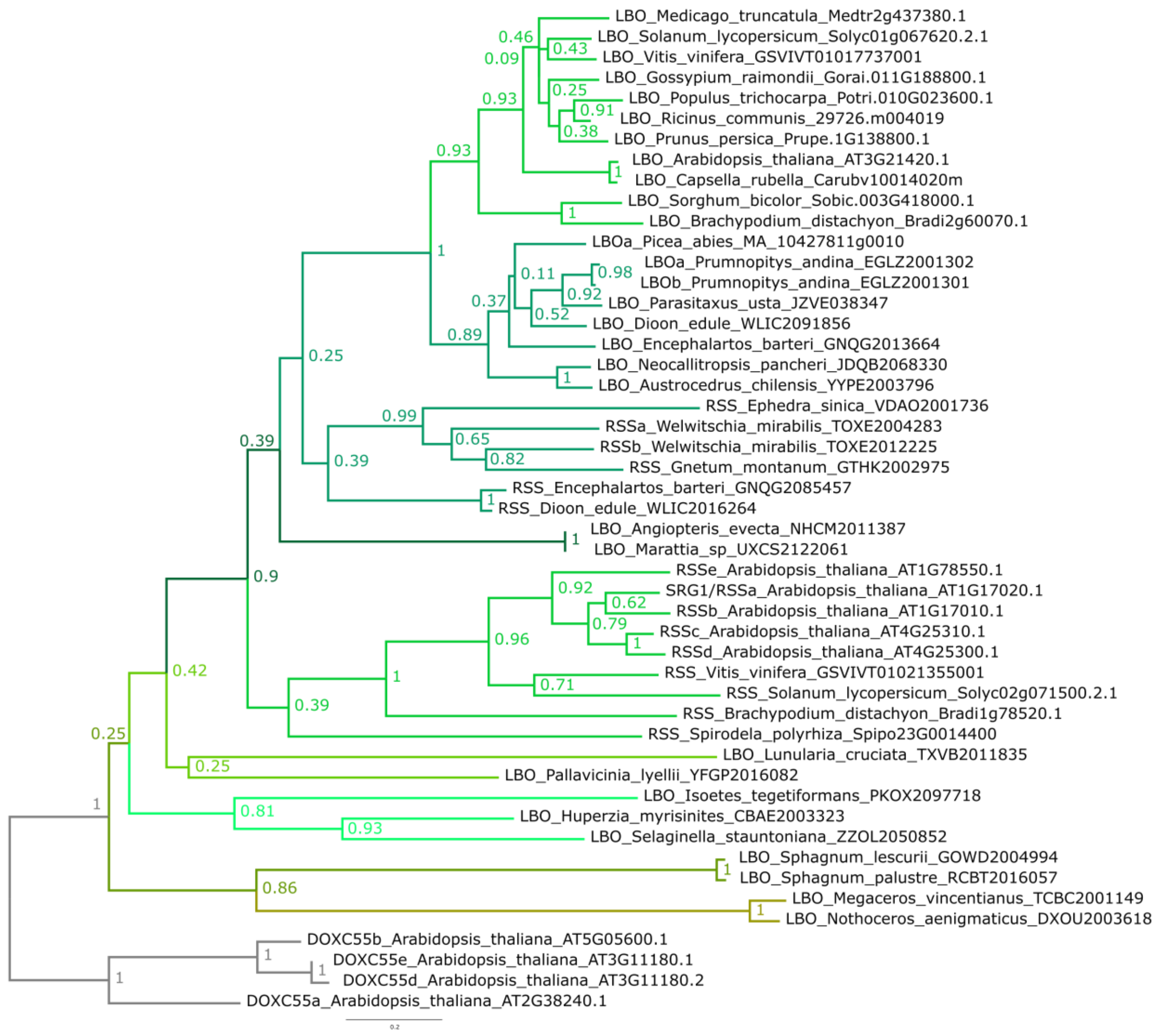

Additional File 13C: Full amino acid-level ML phylogeny for LBO family

Maximum likelihood (ML) tree with the amino acid dataset under the PROTCATLGX model in RAxML. Topology rooted at the DOX55 clade. Bootstrap values are shown at each node of the tree.
