# Supplementary material for: Strigolactone synthesis is ancestral in land plants, but canonical strigolactone signalling is a flowering plant innovation
Source: BMC Biol. 2019 Sep 5;17:70. doi: 10.1186/s12915-019-0689-6 (PMC6728956; doi:10.1186/s12915-019-0689-6)
Supplement: Supplementary file 18 — Table describing final alignments used for phylogenetic analysis. (PDF 9 kb) [file 12915_2019_689_MOESM18_ESM.pdf]

Additional File 18

Gene family	Sequence number	Length	
		Nucleotide	Amino acid
D27	44	1134	197
CCD7	30	3467	537
CCD8	37	2723	474
MAX1	26	1904	505
LBO	50	852	286
SMXL	212	5862	455

Summary of final alignments produced for each gene family studied.
