# Supplementary material for: Strigolactone synthesis is ancestral in land plants, but canonical strigolactone signalling is a flowering plant innovation
Source: BMC Biol. 2019 Sep 5;17:70. doi: 10.1186/s12915-019-0689-6 (PMC6728956; doi:10.1186/s12915-019-0689-6)
Supplement: Supplementary file 19 — Table describing final models used for phylogenetic analysis. (PDF 14 kb) [file 12915_2019_689_MOESM19_ESM.pdf]

## Additional File 19

| | Amino Acid datasets | | | Nucleotide datasets | | Phylobayes Convergence | |
|---|---|---|---|---|---|---|---|
| | **Best model Model Generator** | **Phylobayes model** | **RAxML model** | **IQTree Model (codon)** | **RAxML model** | **Burn-in (Proportion of cycles)** | **Mean diff** |
| **D27** | LG | CAT + LG | PROTCATLGX | MGK+F3X4+R5 | N/A | 0.3 | 0.3 |
| **LBO** | LG | CAT + LG | PROTCATLGX | KOSI07+F+R7 | N/A | 0.3 | 0.3 |
| **MAX1** | LG | CAT + LG | PROTCATLGX | KOSI07+FU+R4 | N/A | 0.3 | 0.3 |
| **CCD7** | LG | CAT + LG | PROTCATLGX | KOSI07+FU+R5 | N/A | 0.3 | 0.3 |
| **CCD8** | LG | CAT + LG | PROTCATLGX | KOSI07+F+R6 | N/A | 0.3 | 0.3 |
| **SMXL** | JTTq | CAT + JTT | PROTCATJTTX | N/A | GTR+Gamma | 0.3 | 0.3 |

Summary of best fitting substitution models employed in each of the phylogenetic analyses and parameters used to assess convergence in Bayesian Inference using Phylobayes. See Methods for further details. The automatic shutoff option was used in Phylobayes which runs bpcomp every 100 cycles until a mean diff of </=0.3 is reached after discarding a burn in of 0.3.
